# Tagging of Endogenous BK Channels with a Fluorogen-Activating Peptide Reveals β4-Mediated Control of Channel Clustering in Cerebellum

**DOI:** 10.3389/fncel.2017.00337

**Published:** 2017-10-31

**Authors:** Christopher P. Pratt, Dika A. Kuljis, Gregg E. Homanics, Jianjun He, Dmytro Kolodieznyi, Srikanth Dudem, Mark A. Hollywood, Alison L. Barth, Marcel P. Bruchez

**Affiliations:** ^1^Department of Biological Sciences, Carnegie Mellon University, Pittsburgh, PA, United States; ^2^Molecular Biosensor and Imaging Center, Carnegie Mellon University, Pittsburgh, PA, United States; ^3^Center for the Neural Basis of Cognition, Carnegie Mellon University, Pittsburgh, PA, United States; ^4^Department of Anesthesiology, University of Pittsburgh, Pittsburgh, PA, United States; ^5^Department of Neurobiology, University of Pittsburgh, Pittsburgh, PA, United States; ^6^Department of Pharmacology and Chemical Biology, University of Pittsburgh, Pittsburgh, PA, United States; ^7^Department of Chemistry, Carnegie Mellon University, Pittsburgh, PA, United States; ^8^Smooth Muscle Research Centre, Dundalk Institute of Technology, Dundalk, Ireland

**Keywords:** BK channels, CRISPR/Cas9, fluorogen-activating peptides, knock-in, Purkinje cells, fluorescent dyes, trafficking

## Abstract

BK channels are critical regulators of neuronal activity, controlling firing, neurotransmitter release, cerebellar function, and BK channel mutations have been linked to seizure disorders. Modulation of BK channel gating is well characterized, regulated by accessory subunit interactions, intracellular signaling pathways, and membrane potential. In contrast, the role of intracellular trafficking mechanisms in controlling BK channel function, especially in live cells, has been less studied. Fluorogen-activating peptides (FAPs) are well-suited for trafficking and physiological studies due to the binding of malachite green (MG)-based dyes with sub-nanomolar affinity to the FAP, resulting in bright, photostable, far-red fluorescence. Cell-excluded MG dyes enable the selective tagging of surface protein and tracking through endocytic pathways. We used CRISPR to insert the FAP at the extracellular N-terminus of BKα in the first exon of its native locus, enabling regulation by the native promoter elements and tag incorporation into multiple splice isoforms. Motor coordination was found to be normal; however, BK channel expression seems to be reduced in some locations. Alternate start site selection or post-translational proteolytic processing resulted in incomplete FAP tagging of the BKα proteins in brain tissues. In Purkinje cell somata, FAP revealed BK channel clustering previously only observed by electron microscopy. Measurement of these clusters in β4^+/-^ and β4^-/-^ mice showed that puncta number and cluster fluorescence intensity on the soma are reduced in β4^-/-^ knockout animals. This novel mouse line provides a versatile fluorescent platform for studying endogenous BK channels in living and fixed tissues. Future studies could apply this line to *ex vivo* neuronal cultures to study live-cell channel trafficking.

## Introduction

The large conductance voltage and calcium-activated potassium channel (BK, MaxiK, Slo1, gene name: *Kcnma1*) is activated by membrane depolarization and elevated intracellular calcium (Toro et al., 1998; Vergara et al., 1998). BK channels are broadly expressed in many tissues including the central nervous system (Tseng-Crank et al., 1994). In the brain, they regulate neurotransmitter release and control neuronal spike rates (Hu et al., 2001; Gu et al., 2007; Contet et al., 2016; Griguoli et al., 2016). The functional channel is formed by a tetramer of α subunits (Toro et al., 1998); alternative splicing produces channels with varied gating properties (Shipston et al., 1999; Soom et al., 2008; Poulsen et al., 2009), responses to intracellular signaling pathways (Tian et al., 2003; van Welie and du Lac, 2011; Toro et al., 2013), and trafficking (Ma et al., 2007; Chiu et al., 2010). BKα knockout mice most starkly show cerebellar ataxia with deficits in coordination, reflex, and spatial learning (Sausbier et al., 2004). In humans, gain or loss of BKα function precipitates epilepsy, developmental delay, and movement disorders (Du et al., 2005; Zhang Z.-B. et al., 2015; Tabarki et al., 2016).

The contribution of a channel to cellular activity depends on its ion flux, which is controlled by the number of channels at the plasma membrane (PM) and their open probability. Mounting evidence suggests that control of trafficking may be a key regulator of BK activity (Toro et al., 2006; Kim E.Y. et al., 2007; Ma et al., 2007; Zarei et al., 2007; Shruti et al., 2012). The relatively low abundance of BK coupled with its high conductance (Kang et al., 1996; Vergara et al., 1998) suggests that modest changes in surface levels could have profound effects on cell activity (Ly et al., 2011). Several kinases acting on BK channels (Wang, 2008; van Welie and du Lac, 2011; Kyle and Braun, 2014) have been shown to affect trafficking of other potassium channels (Connors et al., 2008; Gabriel et al., 2012) and could thus play a role for BK as a long-term form of regulation. Recent work from our lab showed that extended elevation of cyclic AMP with forskolin suppresses surface BKα abundance when expressed alone in heterologous cells (Pratt et al., 2015); a mechanism which could affect BK currents in learning (Nelson et al., 2003; Matthews et al., 2008; Matthews and Disterhoft, 2009).

Up to four β subunits and a single γ subunit assemble with the α tetramer to modify channels’ biophysical properties and trafficking (Behrens et al., 2000; Brenner et al., 2000; Gonzalez-Perez et al., 2014, 2015). The brain-enriched β4 subunit produces BK channels that are resistant to iberiotoxin and charybdotoxin, have slower activation and deactivation rates, and reduced response to calcium (Behrens et al., 2000; Brenner et al., 2000; Lippiat et al., 2003). An endoplasmic reticulum (ER)-retention motif at the C-terminus of β4 regulates the forward trafficking of whole channels in heterologous cells (Shruti et al., 2012), though in the brain this effect appears to be dependent on cell type. CA3 and neocortical pyramidal cells do not exhibit detectable BKαβ4 currents, despite robust expression (Shruti et al., 2012; Cox et al., 2014); however, other neuron types do exhibit these currents, often with β4 localized to specific neuronal compartments (Dopico et al., 1999; Hu et al., 2001; Brenner et al., 2005; Wynne et al., 2009; Martire et al., 2010). In cerebellar Purkinje cells (PCs), the majority of somatic BK current is fluxed through β4-containing channels (Benton et al., 2013), suggesting that β4 may influence BK channel localization and trafficking.

In order to faithfully measure the localization of endogenous BK channels, we generated a gene-targeted mouse in which hemagglutinin (HA) and FAP tag were inserted into at the endogenous BKα locus at exon 1, which is incorporated into all known functional BKα (Erxleben, 2002; Poulsen et al., 2011; Sakai et al., 2011). FAP-BKα channels exhibit similar voltage and calcium responses as untagged BKα. FAP-BKα mice exhibit neither the motor deficits nor aberrant PC pacemaking observed in BKα knockout mice. In the presence of malachite green (MG)-based dyes, FAP produces a bright and highly photostable far-red fluorophore, enabling enhanced tissue penetration and highly stable emission for single-molecule applications. FAP successfully labeled BK channels. While not all α subunits were tagged, likely due to alternate start site selection or post-translational proteolytic processing, tagged and untagged α subunits coassemble into channels. FAP expression in the brain, identified by HA immunostaining and FAP-MG labeling, is representative of native BKα. Using this model, we found that β4 subunit expression is associated with increased clustering of PC somatic BK channels; this characterizes a role for β4 subunit in regulating channel localization beyond ER retention. This mouse model will facilitate future studies of BK channel localization, dynamic trafficking, and single molecule characterization in dissociated neurons and slice cultures.

## Materials and Methods

### Dyes and FAP

Cell-excluded MG-BTau was prepared as described previously (Yan et al., 2015). MG-TCarb, a dye optimized for fixed tissue use and putatively cell excluded, was synthesized using an MG-EDA precursor (Szent-Gyorgyi et al., 2007). The precursor dye was functionalized with a tripod linker containing three *tert*–*butyl* ester functional groups, which were then hydrolyzed to carboxylic acid. Detailed synthesis details can be found in the Supplementary Information. 100–300 μM working stocks (1000×) were prepared by dissolving MG-BTau in PBS; MG-TCarb was dissolved in 95% ethanol to maximize dye stability and solubility. All experiments used the dL5^∗∗^ variety of FAP (Szent-Gyorgyi et al., 2013).

### Cell Culture and Transfection

HEK cells were grown in DMEM/MEM supplemented with 10% heat-inactivated FBS (fetal bovine serum) and 1% penicillin/streptomycin antibiotic at 37°C in a humidified incubator with 5% CO_2_. Wild-type (WT) murine BKα and FAP-BKα encoding plasmid DNAs (accession No. NM_010610.2, available on Addgene, IDs 73212 and 73213) (Pratt et al., 2015) were introduced into HEK cells using the calcium phosphate transfection method. HEK cells were used for electrophysiological recordings 24–48 h after transfection.

### Establishment of FAP-BKα Mouse Line

A targeting plasmid, Cas9 RNA-guided nuclease, and two sgRNAs were used to replace 195 bp of *Kcnma1* exon 1 with an 855 bp insertion containing an N-terminal HA epitope (YPYDVPDYA) and dL5^∗∗^ FAP (Szent-Gyorgyi et al., 2013) fused immediately 5′ of the MDAL start site. An Igκ signal sequence was also used to ensure correct topology; addition of a signal sequence was shown to not disrupt BKα function (Wallner et al., 1996). A cocktail containing sgRNA (12.5–60 ng/μL), Cas9 mRNA (30–110 ng/μL), and undigested circular repair plasmid (100–360 ng/μL) were injected into fertilized C57BL/6J one-cell embryos. A total of 109 founder mice were produced from injected embryos and 102 were analyzed for gene targeting. PCR at the 3′-end showed insertion, but PCR using primers flanking the 5′-end locus was unsuccessful (not shown). Of the screened pups, one founder No. 4159 was identified and mated to C57BL/6J females to establish the FAP-BKα line. This founder mouse was derived from embryos injected cytoplasmically with 50 ng/μL each sgRNA, 100 ng/μL Cas9 mRNA, and 200 ng/μL targeting plasmid. Screening for FAP-BKα-positive mice was initially performed using a primer set (F2R8, see Supplementary Material, Supplementary Table S1 for all primer details) to amplify a 388 bp product if transgene is present. Genotyping was performed with forward primer 5′-GCAACATGGCTGTTGATGGGTGTTC-3′ and reverse primer 5′-GTCACCGGTATGATGAGCGCATCC-3′ cycled using 30 s annealing at 60°C for 20–25 cycles to produce a 309 bp band for the WT allele and a 969 bp band for the transgene. FAP-BKα mice were bred to generate WT, heterozygous (Het), and homozygous (Hom) littermates; zygosity was determined by genotyping. Southern blotting was performed with BKα external to the targeting construct with digestion with EcoRV and BglII. See Supplementary Material for more detailed information. FAP-BKα transgenic mice will be available from The Jackson Laboratory (JAX Strain # 031059).

BK β4 knockout mice were maintained in a C57BL/6J background (Brenner et al., 2005; Shruti et al., 2012). β4 KO mice were crossed with FAP-BKα mice to generate double Het mice and FAP-BKα Hom, β4 KO mice. For FAP-BKα/β4KO experiments, FAP-BKα Hom/β4 KO males were crossed with β4 Het females to generate FAP-BKα Het offspring with β4 Het or KO genotypes.

### Electrophysiology

#### Characterization of FAP-BKα

Experiments were performed at 37°C in 140 mM symmetrical K^+^ solutions which contained 140 mM KCl, 10 mM glucose, 10 mM HEPES, and either 1 mM EGTA (for free [Ca^2+^] ranging 100–300 nM) or 1 mM EDTA (for free [Ca^2+^] 1–10 nM) and Ca^2+^ concentrations were confirmed with a Ca^2+^ electrode. All solutions were made up in double distilled, deionized, filtered water from a MilliQ water purification system. The pipette solution contained 100 nM free Ca^2+^ as per previous studies (Roy et al., 2012, 2014).

Electrodes were pulled from Corning borosilicate glass (1.5 mm OD × 0.86 mm ID) using a Sutter P-97 pipette puller and were fire polished using a Narishige MF 83 microforge. Pipettes had a resistance of 2–5 MΩ when filled with recording solutions and series resistance was compensated by up to 80%. Standard single-channel patch clamp recording methods were used in the inside-out patch conformation. Voltage clamp commands were delivered via an Axopatch 200A patch clamp amplifier (Axon Instruments) connected to a Digidata 1322A AD/DA converter (Axon Instruments) interfaced to a computer running pClamp software (Axon Instruments). Data were acquired at 100 KHz and filtered at 2 KHz. Patches were held at either -60 mV or -100 mV and depolarized in 20 mV increments to 200 mV. Residual capacitance and leakage currents were subtracted using either a P/4 protocol, or offline by manual leak subtraction.

#### Acute Slice Recordings

Three to four week old animals (FAP-BKα heterozygous, homozygous, and WT littermates) were anesthetized with isoflurane, decapitated, and whole-brains dissected in ice cold artificial cerebrospinal fluid (ACSF) with the following composition (millimolar): 119 NaCl, 2.5 KCl, 2.5 CaCl_2_, 1.3 MgSO_4_, 1 NaH_2_PO_4_, 26.2 NaHCO_3_, and 11 glucose equilibrated with 95% O_2_/5% CO_2_. Sagittal slices (350 μm) were sectioned in ice-cold ACSF using a vibratome (Leica VT1200; Buffalo Grove, IL, United States), then transferred to room temperature ACSF for 30–60 min before mounting on a fixed-stage Olympus microscope equipped for differential interference contrast microscopy. Slices were continually perfused with aerated ACSF using a gravity perfusion system. Cell-attached recordings were acquired using the Multiclamp 700B (Molecular Devices) amplifier and National Instruments acquisition interface. Data were filtered at 3 kHz, digitized at 10 kHz, and collected by Igor Pro 6.0 (Wavemetrics). Loose-patch recordings were performed in voltage-clamp mode using glass electrodes (7–10 MΩ) filled with ACSF. Baseline spontaneous action potential frequency was collected over 5 min in ACSF containing 1% dimethyl sulfoxide. Baseline spike rates were compared among genotypes using one-way ANOVA with Tukey’s multiple comparison test (*P* < 0.05).

Primary somatosensory cortical neuron recordings were performed using coronal brain slices harvested from WT and FAP-BKα expressing neonates (P12–15). Layer 2 pyramidal neurons were targeted visually and whole-cell recordings were performed using glass electrodes filled with K-gluconate internal solution, which was composed of the following (millimolar): 125 K-gluconate, 2 KCl, 0.5 EGTA, 10 HEPES, 4 MgATP, 0.3 GTP. Internal solution also contained Alexa488, which was used to fill recorded cells in order to identify pyramidal neuron morphology. Once a GΩ seal was formed and negative pressure applied to rupture the membrane and enter whole-cell recording conditions, the cell was held in current clamp mode for 5 min before input resistance (*R*_IN_) and resting membrane potential readings were collected. To examine action potential waveform, rheobase current (minimal current to elicit one action potential) was injected for each cell examined. After collecting baseline data, MG-Btau (300 nM, H_2_O vehicle) was applied for 10 min, after which resting membrane parameters and action potential waveforms were re-examined. Finally, slices were washed for 20–30 min with ACSF and cells tested for washout effects. Effects of genotype and MG-Btau on resting membrane potential, input resistance, and rheobase current were tested using a repeated measures two-way ANOVA (*P* < 0.05).

#### Curve Fitting and Statistics

Under our recording conditions at 37°C, the tail currents in 100 nM Ca^2+^ deactivated so rapidly that we were unable to accurately determine the activation of the channels as noted previously (Webb et al., 2015). Consequently, conductance (*G*) was derived from steady-state currents according to Ohm’s law

$$G\,\;=\;\frac{1}{V\,\;-\;E_{k}},\,\;\;\;\;\;\;\;\;\;\;\;\;\;\;\;\;\;\;\;\;\;\;\;\;\;\;\;\;\;\;\;\;\;\;\;\;\;\;\;\;\;\;\;\;\;\;\;\;\;\;\;\;\;\left(1\right)$$

where *E*_K_ = 0 mV in symmetrical [K^+^]. Summary data were expressed as the mean ± SEM. *G–V* relationships were fitted with the Boltzmann equation

$$\frac{G}{G_{\max}\,}\,\;=\;\;\frac{1}{1+e^{\frac{V_{m}-V_{1/2}}{S}}},\;\;\;\;\;\;\;\;\;\;\;\;\;\;\;\;\;\;\;\;\;\;\;\;\;\;\;\;\;\;\;\;\;\;\;\;\;\;\;\;\;\;\;\;\;\;\;\;\;\;\;\;\;\;\;\;\left(2\right)$$

where *V*_1/2_ is the voltage of half-maximum activation, *S* is the slope of the curve, V_m_ the test potential, *G* the conductance, and *G*_max_ the maximal conductance. Data from each patch were normalized to the peak conductance measured in 10 μM Ca^2+^ to obtain *G*_max_. All curves were constrained to the *G*_max_ value obtained in 10 μM Ca^2+^.

### Motor Testing

Balance and motor coordination were tested in juvenile littermate mice (5–7 weeks, four to six males, and four to six females) of each genotype on the accelerating rotarod (Med Associates, Inc., St. Albans, VT, United States). For each trial, the rotational speed increased linearly from 6 to 60 RPM over the first 300 s, then continued at 60 RPM for the subsequent 200 s after which the trial was terminated. Mice were tested with five trials per day over five consecutive days at mid-day. A repeated measures two-way ANOVA was used to test for effects of genotype by sex on rotarod latency to fall over all trials (*P* < 0.05), as well as effects of genotype and sex on average latency to fall on the last day of testing. If a significant effect was detected, Tukey’s multiple comparison test was used to identify significant group differences (*P* < 0.05).

Gaits for five to six littermate mice (*n* = 17 animals, WT: 4× F, 2× M; Het 5× F, 1× M; Hom 5× 4, 1× M. Individual litters were sex-matched) were examined by dipping hindpaws and forepaws in blue and yellow watercolor, respectively (Royal Talens, Apeldoorn, Netherlands), before walking on paper. At least four stride length values were obtained for each animal, excluding beginning/end and stopping points. Differences between footprint and effects of genotype were tested using a one-way ANOVA (*P* < 0.05).

All animals’ weights were recorded at the end of testing. Effects of genotype and sex on weight were analyzed using two-way ANOVA; if a significant effect was detected, the Sidak multiple comparison test was used to identify significant group differences (*P* < 0.05). All values reported are means ± SD, unless otherwise stated.

### Antibodies

Mouse monoclonal antibodies against BKα (L6/60, IgG2a) were obtained from Antibodies Inc.^1^ HA tag was detected in immunofluorescence using a mouse monoclonal antibody (HA.11, IgG1 isotype, Covance). For immunoprecipitation (IP), a rabbit HA antibody was used (Clone C29F4, Cell Signaling No. 3724). Calbindin antibody was acquired from EMD Millipore for immunofluorescence.

### Western Blotting and Immunoprecipitation

#### Tissue Processing

Mice of the indicated genotypes were euthanized by CO_2_ inhalation and cervical dislocation. Tissues were rapidly harvested for lysate preparation or flash frozen in liquid nitrogen for later use. For whole lysates, tissues were lysed in modified RIPA buffer (50 mM Tris–HCl, 150 mM NaCl, 1% Triton-X, 0.1% SDS, 0.5% sodium deoxycholate, 1 mM EDTA, pH 7.4) except for IP samples, which were prepared in IP lysis buffer (50 mM HEPES, 137 mM NaCl, 1% Triton-X, 1% sodium deoxycholate, 5 mM EDTA, pH 7.4, 1 mL/100 mg tissue). For crude membrane lysates, brain hemispheres were lysed in a hypertonic lysis buffer (320 mM sucrose, 5 mM HEPES, pH 7.4). Protease inhibitor cocktail (1:100, Sigma–Aldrich) and 1:1000 freshly prepared saturated phenylmethylsulfonyl fluoride (PMSF) were added to all lysis buffers prior to homogenization. Samples were homogenized using a Dounce homogenizer on ice and centrifuged at 900 × *g* to pellet debris and nuclei. For crude membrane preps, supernatant was centrifuged at 100,000 × *g* to pellet membranes; pellet was resuspended in lysis buffer and diluted in SDS sample buffer. Protein was quantified using Bradford and bicinchoninic acid (BCA) assays.

#### Western Blotting

Thirty to fifty micrograms protein from whole lysates and 10 μg from membrane preps were loaded per well on 7% or 4–15% gradient (Bio-Rad) polyacrylamide gels after heating at 37°C for 20 min. Prepared whole lysates of BKα KO brains were generously provided by Dr. Andrea Meredith. Proteins were blotted onto 0.2 μm PVDF membranes using Tris-glycine buffer containing 10% ethanol. Chemiluminescent detection was performed with Immobilon ECL substrate (EMD Millipore) and imaged using a ChemiDoc Touch imaging system (Bio-Rad). Molecular weight analysis and densitometry were performed using ImageLab software (Bio-Rad).

#### Immunoprecipitation

Immunoprecipitation antibodies (rabbit anti-HA monoclonal, mouse anti-BKα, clone L6/60) were added to 500 μL pre-cleared lysate containing 1 mg protein at a 10 μg/mL concentration and incubated at 4°C on a rotator for 2 h. Pre-washed protein G-coated magnetic beads (Thermo Fisher) were added to the lysates and incubated overnight. Samples were washed 3× in IP lysis buffer and once in ultrapure H_2_O. Protein was eluted by incubation in SDS sample buffer containing 2% 2-mercaptoethanol, 50 mM dithiothreitol at 37°C. Samples were analyzed by Western blot as described.

### Image Acquisition and Analysis

#### Cryosectioning

Mice were euthanized by isoflurane overdose and fixed by transcardial perfusion of 0.1 M PBS followed by 4% paraformaldehyde. Brains were removed and post-fixed for 12–24 h at 4°C. Brains were washed in PBS and cryoprotected by 36–48 h incubation in 30% sucrose in PBS, or until brains sank in solution. Frozen sections (30 μm) were cut on a dry-ice cooled cryostat (Leica VT1200; Buffalo Grove, IL, United States) and stored in PBS before histochemical labeling.

#### Immunofluorescence and MG Labeling of FAP

For immunostaining, free-floating sections were permeabilized with 0.1% Triton-X in 0.1 M PBS for 20 min and were then blocked in primary blocking buffer (10% normal goat serum, 2% bovine serum albumin, 0.05% Triton-X) for 30 min. A second blocking step was performed using 0.125 mg/mL goat F(ab) against mouse IgG diluted in primary blocking buffer to block endogenous IgG and Fc receptors. Sections were incubated overnight in indicated antibodies (1:500 BK L6/60, 1:500 HA.11, 1:1000 calbindin). Samples were washed 3× 10 min in PBS containing 0.05% Triton-X and incubated for 1–2 h in 4 μg/mL secondary antibodies (Alexa568-conjugated goat anti-mouse, Alexa488-conjugated goat anti-rabbit for single stains. Alexa568-conjugated anti-mouse IgG2a and Alexa633-conjugated anti-mouse IgG1 were used for BKα and HA co-staining). Secondary antibody was washed out 3× 10 min. Samples were incubated for 20 min with 100 nM MG-TCarb or MG-BTau where indicated, concurrently with 0.8–1.6 μM Hoechst 33342. Three, 5 min washes were performed to remove excess dyes. Slices were mounted using SlowFade Diamond (Thermo Fisher) and imaged on a Zeiss 880 laser scanning confocal or Nikon spinning disk confocal (Andor Technologies, **Figure 6G**).

#### Image Processing

Lipofuscin-like fluorescence was identified by detection with 670 long-pass under 488 or 514 nm excitation due to its broad excitation and emission spectra. Lipofuscin fluorescence was masked in all channels prior to analysis using Imaris for 3D images and Fiji for 2D images. Deconvolution when applied was performed using a 3D-blind algorithm in NIS Elements (Nikon). Low dynamic range channels were smoothed with a 3 × 3 median or Gaussian filter of the approximate point-spread function half width (0.232 μm, approximately one pixel radius).

Purkinje cell dendritic fluorescence was measured in 2D sections by measuring mean molecular layer signal normalized to granule layer signal (*n* = 10 fields from 1 WT, 16 fields from 2 FAP-BKα Het, 18 fields from 2 FAP-BKα Hom mice). PC soma fluorescence was measured in 2D optical sections using ImageJ by calculated total cell fluorescence (CTCF), where

CTCF = Int. Density−(Area⁢∗Background).

Background was measured by taking ROIs in the granule cell layer, which lacks BKα. For analysis, 16 somata from 1 WT, 17 somata from 2 FAP-BKα Het, 17 somata from 2 FAP-BKα Hom mice were used.

Analysis of PC puncta brightness and abundance was performed using a semi-automated algorithm using the spots function of Imaris. For β4 experiments, images were acquired from lobes 7 and 8 of the cerebellum of FAP-BKα Het mice. Because Imaris regions of interest are cubic but PC somata are not, identified spots outside the cell of interest were excluded from analysis. Image IDs were blinded for analysis.

## Results

**FIGURE 1:**
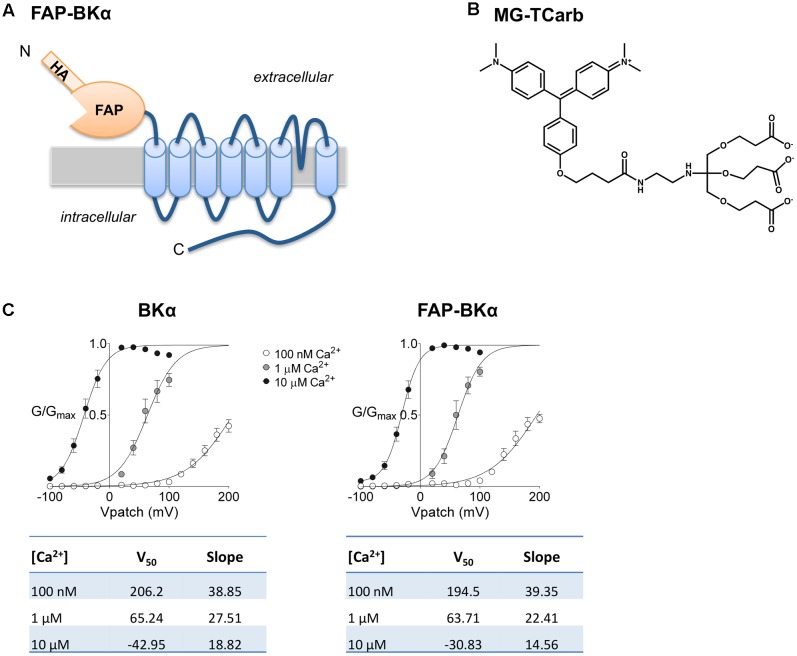
Design of FAP-BKα and MG-TCarb. **(A)** Peptide layout and membrane topology of FAP-BKα construct. **(B)** Structure of MG-TCarb dye. Activation by FAP binding produces fluorescence with 633 nm excitation and 668 nm emission. **(C)** Voltage response of FAP-BKα in varying concentrations of Ca^2+^ compared to the same isoform lacking N-terminal HA/FAP tag. Quantification of V_50_ and Hill slope are shown below for each construct.

Fluorogen-activating peptides have been extensively used to study protein trafficking in cultured cells (Shruti et al., 2012; Pratt et al., 2015; Yan et al., 2015), but *in vivo* use is still in its infancy (Wang et al., 2015, 2017; Zhang M. et al., 2015; He et al., 2016). FAP yields a bright and highly photostable, far-red fluorophore, with a molecular brightness that surpasses other genetically encoded red fluorescent proteins (Saurabh et al., 2016). An N-terminally tagged FAP-BKα construct was created to avoid interfering with C-terminal trafficking motifs and to enable surface-exclusive labeling (**Figure 1A**); this design was previously described for BKα overexpression experiments in cultured cells (Pratt et al., 2015). With existing MG variants, including cell-excluded MG-BTau (Yan et al., 2015), we observed non-specific targeting of dead and dying cells. To reduce this effect, and for optimal labeling in fixed tissues, we designed the fluorogen MG-TCarb (**Figure 1B**). Several dyes were designed with negatively charged moieties to inhibit DNA intercalation; the tripod configuration of MG-TCarb showed the lowest background and nuclear staining with the highest signal to noise of dyes tested. Electrophysiological measurements of FAP-BKα cDNA in Human embryonic kidney 293 (HEK293) cells showed similar voltage and calcium responses to the untagged version (**Figure 1C**), indicating that the FAP tag does not interfere with the biophysical properties of BKα.

**FIGURE 2:**
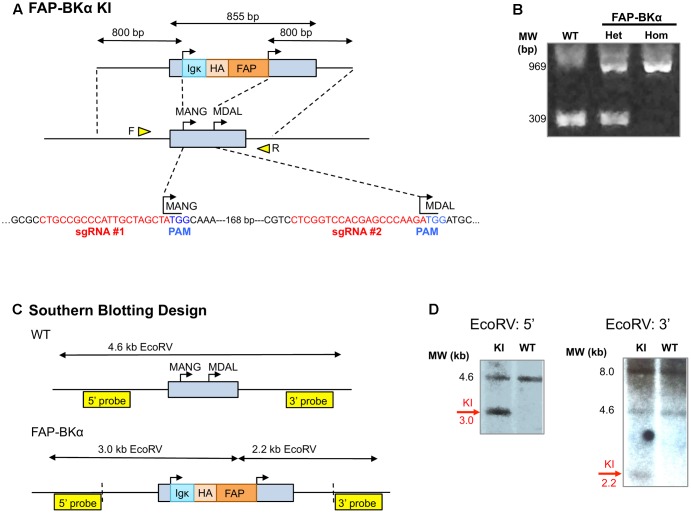
CRISPR-mediated gene targeting. **(A)** Schematic for CRISPR/Cas9 targeting of *Kcnma1* exon 1, showing knock-in (KI) insertion construct. The native *Kcnma1* allele is shown below; targeting removed the first start codon (MANG-) and inserted FAP upstream of the downstream start codon (MDAL-). sgRNA sequences for CRISPR targeting are shown in red with protospacer adjacent motifs (PAMs) shown in blue. Primer locations for allele-specific PCR are depicted as yellow triangles. **(B)** Allele-specific PCR of WT, FAP-BKα het and homozygous mice yields expected band sizes at 309 and 969 bp, respectively, for WT and KI alleles. **(C)** Schematic for Southern blot analysis of KI using EcoRV digestion with 3′ and 5′ external hybridization probes. Vertical dashed lines indicate insertion boundaries. **(D)** Southern blots digestion showed the expected sizes for WT and knock-in (KI) alleles with EcoRV digestion. KI-specific signal is indicated.

Using CRISPR, we aimed to express tagged channels that would maintain native expression levels, across multiple splice isoforms. This approach is key due to the extensive and tissue-specific alternative splicing in BKα (Shipston et al., 1999; Ma et al., 2007; Poulsen et al., 2009; Kyle and Braun, 2014) and controlled stoichiometry with accessory subunits. To that end, a DNA-targeting plasmid and the Cas9 RNA guided nuclease and two sgRNAs were used to insert a FAP cassette into the native BKα locus, *Kcnma1* (**Figure 2A**). PCR spanning the targeted locus using primers flanking the first exon of *Kcnma1* showed the expected insertion size (**Figure 2B**); loss of the WT allele in FAP-BKα Hom demonstrated that FAP-BKα and native BKα are alleles in the same genomic location. Southern blotting with BKα probes not present on the targeting construct showed hybridization to the expected EcoRV digestion fragments (**Figures 2C,D**). However, hybridization to BglII-digested DNA revealed a larger-than expected fragment (Supplementary Figure S1). The reason for this was undetermined, as sequencing attempts through this locus with high complexity were unsuccessful.

### FAP-BKα Mice Do Not Exhibit Deficits in Motor Coordination

**FIGURE 3:**
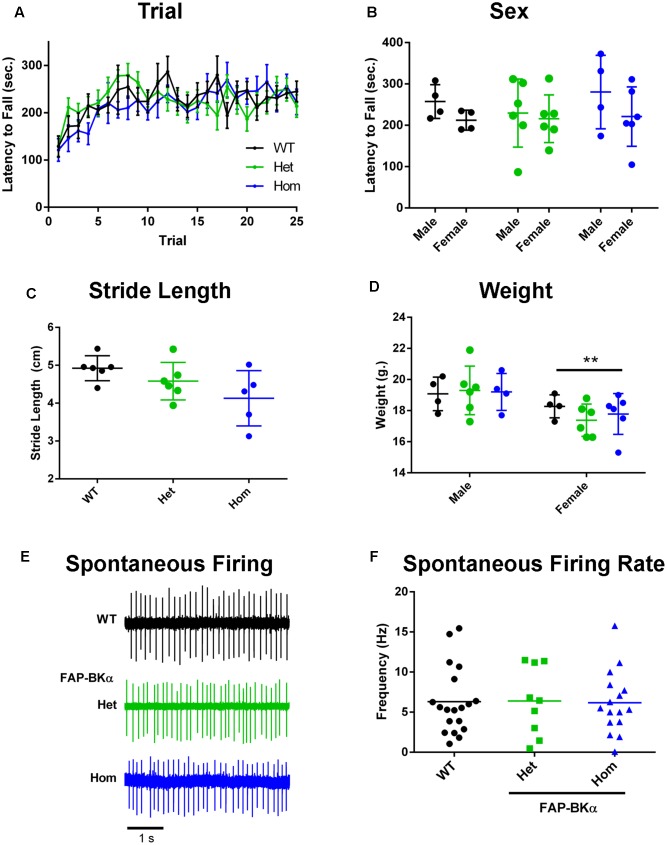
Measurements of FAP-BKα motor effects and PC physiology. **(A)** Performance on the accelerating rotarod (6–60 × *g* over 300 s, 200 s sustained) segregated by genotype and trial number. **(B)** Performance on accelerating rotarod based on sex. **(C)** Measurements of stride length of WT, FAP-BKα Het and Hom littermates. **(D)** Measurements of mouse weight segregated by sex and genotype. ^∗∗^*P* ≤ 0.01 based on sex (*n* = WT F: 4, M: 2; Het F: 5 F, M: 1; Hom F: 4, M: 1) **(E)** Example traces from spontaneously firing PCs in acute slices from WT, FAP-BKα Het, and FAP-BKα Hom mice. **(F)** Quantification of spontaneous spike rate in WT, FAP-BKα Het, and FAP-BKα Hom mice (*n* = WT: 4 mice, 19 PCs; Het: 4 mice, 9 PCs; Hom: 3 mice, 16 PCs).

To verify that FAP does not render BKα non-functional *in vivo* and thus impose a dominant-negative phenotype, we asked if FAP-BKα expressing mice would phenocopy BKα null mice. BKα knockout mice exhibit profound ataxia and changes in gait (Sausbier et al., 2004; Chen X. et al., 2010), a phenotype also produced by cerebellar microinjection of paxilline, a potent BK channel antagonist (Cheron et al., 2009). To determine if FAP-BKα mice exhibited any of these deficits, WT and transgenic performance was compared using the accelerating rotarod. No differences were observed based on genotype or sex (**Figures 3A,B**, detailed statistics in **Table 1**). When gait was analyzed by footprint analysis of stride length, we found no significant differences across genotypes, with the majority of variation coming from individual mice (**Figure 3C** and **Table 2**). It is possible that further testing could reveal an effect of FAP-BKα on stride length; however, this effect would seem to be subtle. Because BKα knockout mice show reduced body weight (Sausbier et al., 2004), and because weights could affect stride length, mouse weights were recorded (**Figure 3D**). As expected, male mice weighed more than their female counterparts; however, no significant effect based on genotype was observed (**Table 2**). The range of mouse weights appeared to have little bearing on the stride length (Supplementary Figure S2), and short stride length did not appear to associate with poor measures of coordination.

**Table 1:** Summary statistics for Rotarod tests.

	SS	MS	*df*	*F*	*P*
**Rotarod performance (trial × genotype)**					
Interaction	267,071	5564	48	0.9917	0.4914
Trial	563,296	23,471	24	4.183	<10^-4^
Genotype	13,443	6722	2	0.1799	0.8364
Subjects (matching)	1.01^∗^10^6^	37,372	27	6.661	<10^-4^
**Final day Rotarod performance (sex × genotype)**					
Interaction	2969	1484	2	0.3332	0.7199
Sex	11,145	11,145	1	2.502	0.1268
Genotype	4165	2083	2	0.4675	0.6322


*Summary statistics for two-way ANOVA tests of Rotarod performance are detailed. Rotarod results were quantified by genotype and trial as well as by sex and genotype on the final day of testing. SS, sum of squares; MS, mean of squares; df, degrees of freedom; F and P statistics are shown*.

**Table 2:** Summary ANOVA statistics for stride length and weight measurements.

	SS	MS	*df*	*F*	*P*
**Stride length (one way)**					
Genotype	1.723	0.8614	2	3.090	0.0773
Individual	3.903	0.2788	14		
**Weight (sex × genotype)**					
Interaction	1.501	0.7505	2	0.5063	0.6090
Sex	13.67	13.67	1	9.221	0.0057
Genotype	0.5351	0.2676	2	0.1805	0.8360


*ANOVA statistics for stride length and weight are shown. SS, sum of squares; MS, mean of squares; df, degrees of freedom; F and P statistics are shown*.

Lastly, we assessed BK channel function in PCs by measuring their spontaneous firing rates in acute slices. Previous work has demonstrated a dependence of spike rate on functional BK channels, in which genetic ablation reduced spike rate (Sausbier et al., 2004; Cheron et al., 2009). We found no difference in spike rate across littermates of the three genotypes [**Figures 3E,F**, mean firing rate 6.25 Hz; one-way ANOVA genotype, *F*_(2,41)_ = 0.008, *P* = 0.99]. Treatment with BK channel blockers has also been shown to affect spike rate (Edgerton and Reinhart, 2003); however, addition of 10 μM paxilline for 10 min did not induce any consistent changes in spike rate (data not shown). Unlike previous studies, our experiments were performed at room temperature; it is unknown if increasing temperature would lead to a discernible phenotype or effect of paxilline. We concluded that the FAP-BKα transgene does not influence gross motor coordination, nor does it have overt effects on PC spontaneous firing. Overall, we did not observe any phenotype consistent with non-functioning BK channels.

### FAP-Tagged BKα Subunits Comprise a Subpopulation that Coassembles with Untagged BKα

**FIGURE 4:**
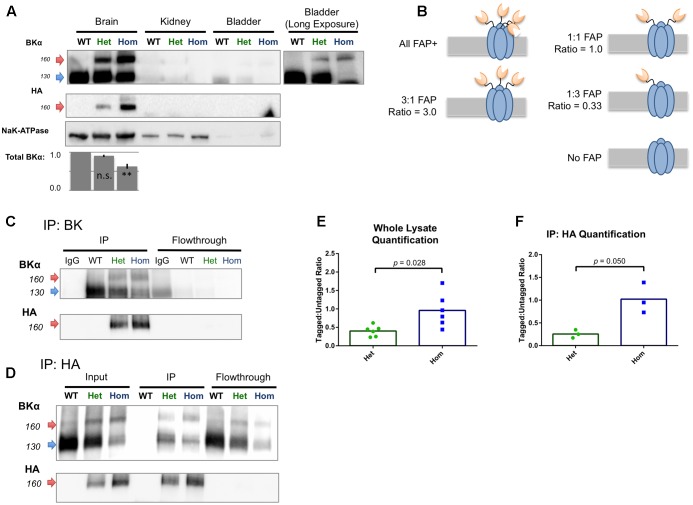
FAP-tagged BKα subunits coassemble with untagged subunits. **(A)** Crude membrane fractions from brain, kidney, and bladder were probed for BKα and HA tag. Untagged BKα migrated at 125 kDa and FAP-BKα migrated at 155 kDa. Both bands were present in brain, with HA reactivity in the 155 kDa band. To visualize weak signal in bladder smooth muscle, a long exposure is shown; however, HA reactivity was undetectable in these preparations. Quantification of total BKα normalized to NaK-ATPase is shown below. *n* = 2 biological replicates, error bars are SD. ^∗∗^*P* ≤ 0.01 compared to WT, one-way ANOVA with Tukey’s multiple comparison test. **(B)** Schematic for five possible configurations of BKα:FAP-BKα tetramerization ranging from homomeric FAP-BKα to channels lacking FAP; expected densitometry ratios are given for each condition. **(C)** Immunoprecipitation using antibodies directed against BKα C-terminus yielded FAP-tagged and untagged channels. FAP-tagged channels showed HA reactivity, which was depleted in flowthrough. **(D)** Immunoprecipitation using antibodies against HA yielded tagged and untagged channels. Only the high M.W. bands were HA reactive in input and IP. **(E,F)** Ratios of high and low (155 kDa:125 kDa) BKα-reactive band densitometry in whole lysates (**E**, *n* = 4 animals, 6 protein preparations) and HA immunoprecipitated samples (**F**, *n* = 2 animals, 3 protein preparations). Significance tested using unpaired *t*-test with Welch’s correction.

Protein lysates prepared from whole adult mouse tissues of all three genotypes were analyzed by Western blotting; crude membrane fractions were prepared from BKα expressing whole brain, kidney, and bladder of WT and knock-in animals and probed for BKα and HA immunoreactivity (**Figure 4A**). In WT brain, BKα immunoreactivity was observed at the expected molecular weight of 125 kDa, with a non-specific band at about 105 kDa. In FAP-BKα expressing mice, an additional band was observed at approximately 155 kDa, a molecular weight shift that corresponded to the HA-FAP insertion. FAP-BKα Hom showed a heavier upper band with a narrowed lower band. Only the upper bands were reactive for HA, suggesting that indeed these are tagged with FAP. No BKα was detected in kidney membranes, this is likely due to BKα expression being primarily localized to the collecting ducts and expressed at fairly low levels unless the animal is fed a high potassium diet (Wen et al., 2013). In bladder, the upper bands appeared as in brain, but in FAP-BKα Hom mice, the lower, untagged band disappeared entirely. No HA was detected in the bladder lysates, likely due to signal being below the detection limit. Quantification of total BKα by summating the 125 and 155 kDa band densities and normalizing to NaK-ATPase in membrane fractions revealed no change in FAP-BKα Het, but significantly reduced total expression in Hom [*n* = 2 mice per genotype. Percentage compared to WT (±SD): Het = 90.4(±3.25), Hom = 62.8(±6.22)].

Western blotting of BKα KO whole brain lysates concurrently with a WT, FAP-BKα Het, and Hom trio confirmed antibody specificity (Supplementary Figure S3A). We concluded that this lower band at 125 kDa indeed results from BKα. Two explanations for the presence of these bands are first, that an alternative translation initiation site can result in skipping of the FAP translation in certain peptides, a phenomenon observed in two other potassium channels (Fernandez et al., 2003; Simkin et al., 2008). A second explanation is that a proteolytic cleavage event could remove the tag from the nascent peptide; in human BKα, cleavage has been reported in the S0–S1 intracellular linker (Korovkina et al., 2006). The blots of crude membrane fractions showed no specific HA reactivity at low molecular weights, nor did whole soluble lysates reveal any HA-reactive peptides greater than 15 kDa (Supplementary Figures S3B,C).

Despite the fact that not all BKα subunits in the brain contain FAP, it is possible that channels could still be tagged by FAP by heterotetramerization of tagged and untagged α subunits. Alternatively, FAP-BKα could preferentially segregate into its own population of channels. In order to examine this, reciprocal IP experiments were performed using antibodies against BKα and HA; BKα antibodies should purify both tagged and untagged channels, discernible by Western blotting. If FAP-bearing channels homotetramerize into their own population, then IP by HA should only yield BKα of the high molecular weight. On the other hand, if heterotetramers form, then IP with HA should yield both tagged, HA reactive BKα, and untagged, HA-negative BKα. The ratio of tagged:untagged BKα can be measured to infer a mean FAP stoichiometry (**Figure 4B**).

BK IP, as expected, yielded both tagged and untagged channels, similarly to input lysates (**Figure 4C**). Performing the IP protocol on WT lysates using a mouse HA antibody as an IgG control failed to yield any bands. BKα and HA signal were successfully depleted from flowthrough, demonstrating an efficient capture of BKα and that HA specificity is BKα-dependent. IP using an HA rabbit monoclonal antibody yielded both tagged and untagged subunits (**Figure 4D**) in which only the upper, 155 kDa bands were HA reactive. IP of WT lysates produced no bands. However, faint bands of approximately 155 kDa remained in the flowthrough that did not show HA reactivity in WB. The identity of these bands is unclear.

We measured the ratio of tagged:untagged channels to characterize the relative abundance of the FAP-tagged variant in whole lysates and HA IPs. In raw lysates, FAP-BKα Het mice had a mean ratio of 0.40 (±0.06 SEM, *n* = 6) and FAP-BKα Hom showed a mean ratio of 0.96 (±0.19 SEM, *n* = 6) (**Figure 4E**). This shows roughly a dose-dependent effect of genotype on FAP-tagged BKα abundance. With HA IP, FAP-BKα Het shows a ratio of 0.26 (±0.05, *n* = 3) and Hom showed 1.02 (±0.19, *n* = 3) (**Figure 4F**). We concluded that FAP-BKα Het mice generally contain 1 FAP per assembled channel, but not all channels are labeled, resulting in a decrease in ratio following IP as well as persistence of BKα in flowthrough. FAP-BKα Hom mice have 2 FAPs per channel, again showing the dose-dependence of the genotype on FAP tagging, and this remains remarkably consistent with the whole lysate. However, as in Het mice, Hom also does not tag every channel in the brain.

### FAP and BKα Localize to the Same Brain Regions, Strong Expression in Cerebellum

**FIGURE 5:**
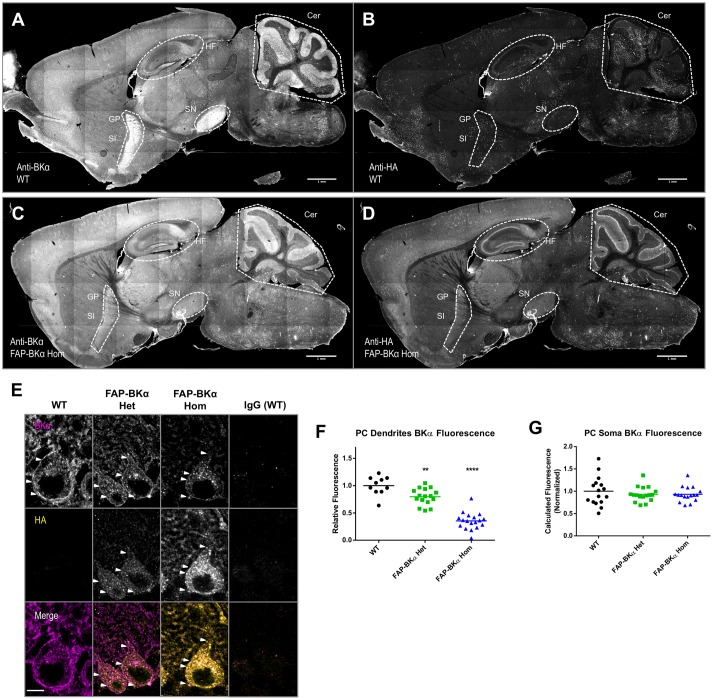
Hemagglutinin (HA) localization corresponds to same brain regions as endogenous BKα; not all BKα in PCs show HA reactivity. Sagittal cryosections of p60 WT **(A,B)** and FAP-BKα homozygous **(C,D)** brains were co-stained for BKα **(A,C)** and HA **(B,D)**. BKα shows notably strong expression in hippocampal formation (HF), cerebellum (Cer), substantia nigra (SN). Expression is also evident in Substantia innominata (SI) and globus pallidus (GP), which appear as a continuous region. **(B)** HA staining does not produce specific signal in WT. **(D)** FAP-BKα Hom mice show localization of HA signal to be similar to BKα. FAP-BKα heterozygous brain is not shown due to insufficient signal-to-noise in whole brain under immunostaining conditions. **(E)** Confocal sections of PCs show BKα expression in somata and dendrites in the PC and molecular layers, respectively. HA immunoreactivity is more strongly localized to soma than total BKα. White arrowheads point to example local maxima showing BKα and HA signal. **(F)** Quantification of total BKα fluorescence in PC dendrites (each point corresponds to a field of view from two to three animals of each genotype, normalized to granule cell layer fluorescence). **(G)** Total calculated cell fluorescence for PC somata, background calculated from granule cell layer. ^∗∗^*P* ≤ 10^-3^, ^∗∗∗∗^*P* ≤ 10^-5^ compared to WT.

For studying BK in the brain, it is necessary to know if FAP-BKα is expressed in the correct cells and subcellular domains to recapitulate native BKα. BK channels are critical for cerebellar function, and are abundantly expressed. Using mouse monoclonal antibodies against BKα and HA, immunofluorescence staining was performed in sagittal cryosections from WT (**Figures 5A,B**) and FAP-BKα Hom mice (**Figures 5C,D**). BKα staining showed a broad expression pattern, with especially stark expression in the cerebellum, hippocampus, globus pallidus, substantia innominata, substantia nigra, and other regions, although BKα intensity appears to be reduced in some areas. HA staining showed no signal above background in WT, but showed signal mirroring that of BKα in the FAP-BKα Hom. Interestingly, BKα staining intensity was reduced in several areas including substantia nigra and globus pallidus–substantia innominata. FAP-BKα Het brains did not yield acceptable signal-to-noise with HA staining in most brain regions (not shown). The HA staining showed signal in the granule cell layer of dentate gyrus and stratum pyramidale in CA3-CA1. Given that our BKα staining shows an absence in stratum pyramidale, as do previously published immunohistochemistry experiments in rat hippocampus using the same antibody (Misonou et al., 2006), and that a weak signal can also be seen in the WT brain, we interpret this as non-specific signal.

Confocal imaging of cerebellar PCs confirmed strong immunolocalization to PC somata, where a strong putative PM signal is evident, with visible local maxima. As previously observed (Kaufmann et al., 2009) there is moderate intensity in the molecular layer, which contains PC dendrites (**Figure 5E**). One unexpected result came as a loss in dendritic immunoreactivity in FAP-BKα knock-in animals (**Figure 5F**, relative dendritic intensity compared to WT: Het = 80%, Hom = 35%. *n* = 10 fields from 1 WT, 16 fields from 2 Het, 18 fields from 2 Hom mice), while total BKα signal in PC somata was unchanged (**Figure 5G**). Together these data show that while immunofluorescence of HA and BKα are expressed in the same cells, detectable FAP-BKα channels are preferentially localized to the PC soma. Furthermore, FAP-BKα expressing mice appear to have deficits in PC dendritic localization; however, the extent of this disruption is FAP-BKα dose dependent. Despite a reduced brightness of signal, the use of FAP-BKα Het mice for MG-TCarb labeling experiments seemed a worthy compromise to visualize channels while minimizing disruption of BKα localization, since both Het and Hom animals express untagged channels.

### MG Labeling of FAP Recapitulates HA Immunolocalization

**FIGURE 6:**
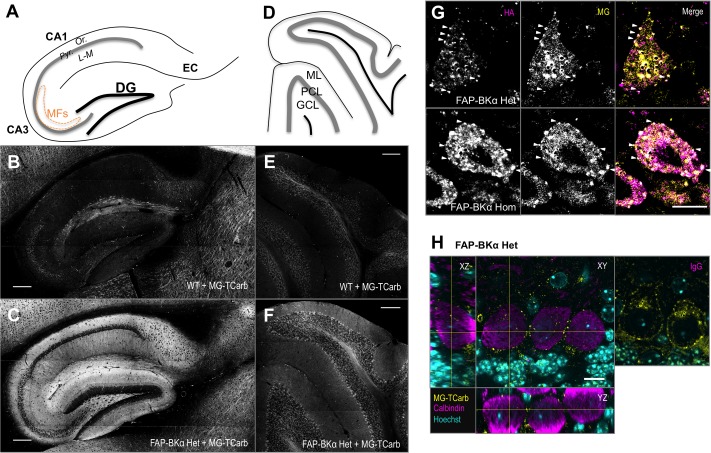
Sagittal sections labeled with MG-TCarb show expected FAP signal in hippocampus and cerebellum. Punctate distribution seen in PCs. **(A)** Schematic of sagittal hippocampal sections depicting the regions of dentate gyrus (DG), CA3, CA1, and entorhinal cortex (EC). CA layers depicted are stratum pyramidale (Pyr.), stratum oriens (Or.), and stratum lacunosum-moleculare (L-M). Mossy fibers (MFs) in CA3 are depicted by the orange dashed line. **(B,C)** Images of WT **(B)** and FAP-BKα Het **(C)** hippocampi showing FAP-BKα dependent fluorogen activation. Scale bars 200 μm. **(D)** Schematic of sagittal cerebellar sections depicting the three main layers: granule cell layer (GCL), Purkinje cell layer (PCL), and molecular layer (ML). **(E,F)** Images of WT **(E)** and FAP-BKα Het **(F)** cerebella showing FAP-BKα-dependent fluorogen activation. Scale bars 200 μm. **(G)** Single optical sections of PCs stained with HA antibody (magenta) and labeled with MG-TCarb (yellow) show colocalization to puncta at the cell periphery, with diffuse signal within the cytoplasmic region in FAP-BKα expressing brains. **(H)** Staining of PC volumes with calbindin (magenta) shows FAP (yellow) puncta are localized to the cell edge in three dimensions. Hoechst 33342 staining (cyan) shows orientation of PC layer with abundant signal in GCL layer below. No calbindin signal is observed in IgG controls. Scale bars 10 μm.

Addition of MG-TCarb produced a fluorescent signal in sagittal posterior brain sections similar to that of BKα and HA immunolocalization, including bright, specific signal in hippocampus and cerebellum. In the hippocampus (**Figures 6A–C**), FAP-BKα is localized to expected regions, with an exclusion from CA3 and CA1 pyramidal cell bodies in stratum pyramidale, but robust expression in stratum oriens, stratum lucidum, the site of mossy fiber connections, and stratum lacunosum-moleculare, where Schaffer collaterals provide synaptic input. This is in agreement with previous reports (Hu et al., 2001; Misonou et al., 2006; Cox et al., 2014). Cerebellum (**Figures 6D–F**) shows strongest FAP signal in PC bodies with moderate fluorescence in the molecular layer, in agreement with BKα immunostaining and previous reports (Misonou et al., 2006; Kaufmann et al., 2009). Signal is dependent on FAP expression and MG addition (Supplementary Figure S4). A low level of non-specific, dye-related fluorescence does occur with MG-TCarb addition to non-FAP expressing brains; however, the presence of FAP-BKα and dye washout prior to slide mounting suppressed this signal substantially.

We next aimed to confirm that MG labeling of FAP recapitulates HA immunostaining. To that end, we had optimized a labeling paradigm using MG-TCarb, which was designed to minimize dead-cell staining and non-specific activation in fixed tissue. Immunostaining against HA and subsequently labeling with MG-TCarb revealed bright puncta in PCs, which were spatially colocalized (**Figure 6G**, white arrowheads). The brightness of these puncta did not always co-vary linearly, though this likely stems from the different labeling and detection methods. Previous reports using electron microscopy and freeze-fracture showed large clusters containing BK channels at the PM of PC somata (Kaufmann et al., 2009; Indriati et al., 2013), to confirm that these puncta are localized to the PM, we immunostained using the cytoplasmic PC-specific marker calbindin (**Figure 6H**), where edges represent putative PM. MG-TCarb puncta were localized to the edge of calbindin-positive areas in all three dimensions of imaging, suggesting that these puncta are likely to be PM localized.

Fluorogen-activating peptides labeling has several advantages over antibody-based detection, especially in live cells, including selectable cell permeance or impermeance, high molecular brightness, and labeling with a small molecule label that does not require wash steps. Various MG derivatives find optimal niches in different applications. MG-TCarb is useful for labeling fixed cryosections because of the reduced non-specific labeling; however, ethanol solvation requirements limit its utility in live cells and sections. In order to prepare this model for future studies, we measured the effects of a cell-excluded dye ideal for live studies, MG-BTau (Yan et al., 2015; Wang et al., 2017), on the electrical properties of neocortical neurons in acute slices. We found that addition of 300 nM MG-BTau did not alter resting membrane potential, input resistance, rheobase current, or AP time-course (Supplementary Figure S5).

### Loss of β4 Impairs Assembly into PM Clusters

The β4 subunit produces BK channels with slower activation and deactivation kinetics and altered sensitivity to calcium (Behrens et al., 2000; Brenner et al., 2000; Lippiat et al., 2003). While β4 has an ER-retention motif (Shruti et al., 2012; Cox et al., 2014), the localization of β4-containing BK channels to specific PM domains (Dopico et al., 1999; Martin et al., 2004; Wynne et al., 2009; Martire et al., 2010) suggests that cell-type-specific mechanisms are involved to direct these channels to specific PM locations. In PCs, BKα/β4 channels carry the bulk of somatic BK current (Benton et al., 2013). Furthermore, clustering of BK into large intramembrane particles along with P/Q type calcium channels has been characterized in PC somata by freeze fracture and EM, with both of these channels being involved in assembly (Kaufmann et al., 2009; Indriati et al., 2013). BK channels also exist in a non-clustered, scattered population. The distinct roles of the clustered and scattered channels are unknown. FAP-BKα effectively reveals these clusters.

**FIGURE 7:**
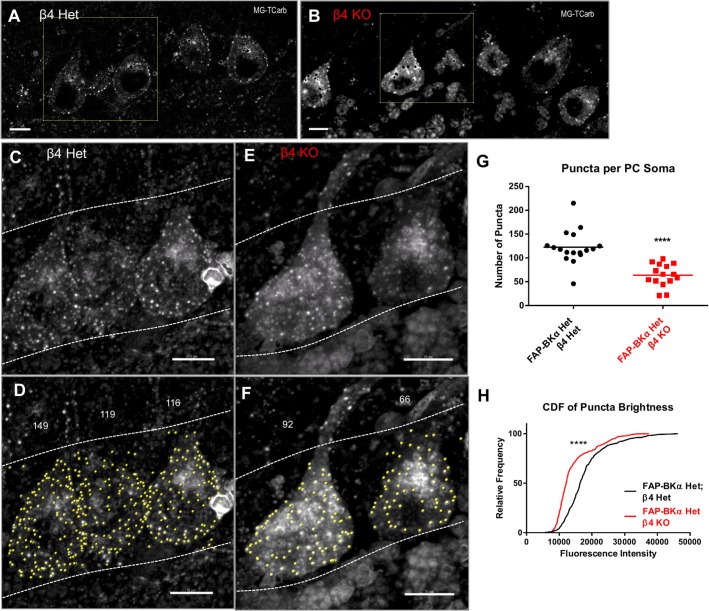
β4 knockout reduces the number of PM FAP-BKα puncta in Purkinje neurons in FAP-BKα Het mice. **(A)** FAP-BKα Het; β4 Het brains show PM puncta in single optical sections. Black spots result from lipofuscin masking operations. The yellow square indicates cells in **(C,D)**. **(B)** β4KO brains show DPM puncta in single optical sections. The yellow square indicates cells in **(E,F)**. **(C)** *Z*-projection of three PC somata showing bright FAP puncta. Dashed lines indicate borders of PCL with GCL below and ML above. **(D)** Identification of spots (yellow) by Imaris yields punctum counts per cell. Stray spots not clearly assigned to a soma were excluded from analysis. Counts for the given PCs are shown above. Dashed white lines show area of analysis, demarcating the PCL boundaries. **(E)** *Z*-projection of β4KO brains show puncta similar to β4 Het. **(F)** Analysis of β4KO PCs performed analogously to **(D)**. **(G)** Puncta counts per cell for β4 Het and β4KO brains. ^∗∗∗∗^*P* ≤ 10^-4^, unpaired *t*-test with Welch’s correction. **(H)** Cumulative distribution function for all counted puncta by genotype. ^∗∗∗∗^*P* ≤ 10^-4^, Mann–Whitney rank sum test.

In order to minimize experimental variation, we opted to use littermate mice that could be processed in parallel. To this end, we crossed FAP-BKα Hom mice with β4 KO mice to generate FAP-BKα Het mice with β4Het or KO genotypes. FAP-BKα puncta are readily visible at the cell periphery in the PC layer in single sections (**Figures 7A,B**). The intensity of diffuse FAP signal does not appear to be an effect of genotype, as cells high in internal fluorescence have also been observed in β4 Het tissues, and conversely cells with low internal fluorescence have been observed in β4 KO tissues (not shown). 3D images were generated from acquired stacks (**Figures 7C,D**); only cells in which the entire soma is included in the stack were used for quantification. The genotypes were blinded prior to analysis, and unblinded after quantification. Max projection images of whole somata were constructed and cells were analyzed by spots detection using Imaris (**Figures 7E,F**); the numbers of puncta per PC soma were counted (**Figure 7G**, mean number of puncta ± SEM: β4 Het: 122.7 ± 8.55, β4 KO: 63.73 ± 6.15, *P* ≤ 10^-4^, unpaired *t*-test with Welch’s correction. *n* = 17, 15 cells for β4 Het and β4 KO, respectively, from 2 male animals per genotype). The puncta brightness was also quantified per spot as the median MG voxel intensity within the spot. The distribution of puncta brightness is significantly reduced in the β4 knockout (**Figure 7H**). Together, these data indicate that loss of β4 reduces the number of membrane puncta present in PCs by nearly a factor of two. Additionally, based on fluorescence intensity, the average number of channels incorporated into each punctum is also reduced. This approach only allowed us to examine the effects of total loss of β4 on clustering, since our breeding strategy resulted in a lack of β4 WT mice. We hypothesize that the assembly of clusters could be further pronounced in β4 WT animals, but the necessary stoichiometry of β4 to exert a trafficking effect is unknown.

## Discussion

BK channels are critical determinants of cell activity, enabling rapid firing through their strong repolarizing current and rapid deactivation. BK is especially important in PCs for enabling complex spikes, which are critical for reflex learning and motor coordination. Mice lacking BKα channels are viable; however, they suffer from cerebellum-based motor deficits (Sausbier et al., 2004; Chen X. et al., 2010). Knocking out β4 is sufficient to produce seizure activity, but mice do not have measurable motor deficits (Brenner et al., 2005). The observed effects of β4 loss may be based both on trafficking and kinetic alterations of BK currents; in dentate gyrus, which possess surface BKαβ4 channels, loss of β4 enhances excitability by enhancing coupling to internal calcium stores and through increased BK activation and deactivation speeds (Brenner et al., 2005; Wang et al., 2016). In the CA3 region of the hippocampus, where the β4 ER retention motif effectively sequesters BK channels, β4 knockout enhances surface BK abundance, facilitating rapid spiking by increasing BK current flux. Biophysical studies using these knockouts, specific inhibitors, and electrophysiology have yielded great knowledge at the general role of BK channels, as well as some of the protein-level effects of signaling, β subunit incorporation, and alternative splicing. However, these experiments only tell a part of the story.

We generated the FAP-BKα mouse, the first genetically engineered animal in which BKα is tagged with a detectable substrate, which enables the visualization and study of BK channels under endogenous control of expression and splicing. FAP-BKα failed to label all channels in the brain; however, FAP-BKα appears to have similar localization properties to native BKα. In PC somata, however, FAP-BKα strongly visualized channel clusters previously seen only using electron microscopy (Kaufmann et al., 2009; Indriati et al., 2013). Like these previous studies, BK-containing clusters were limited to the soma and proximal dendrite; the reason for their enhanced fluorescence could lie in the different detection methods of using FAP-MG labeling versus immunofluorescence. Importantly, FAP-BKα homomers appear to retain voltage and calcium sensitivity in heterologous cells, and FAP-BKα Het and Hom mice do not appear to have gross motor deficits or seizures, symptoms of BKα loss or gain of function (Sausbier et al., 2004; Shruti et al., 2008; Chen X. et al., 2010). We did not observe any change in PC spontaneous firing; however, the lack of an effect of paxilline on output is concerning. It is possible that our approach at room temperature in acute slices does not reproduce BK-dependent effects previously described (Edgerton and Reinhart, 2003; Sausbier et al., 2004; Cheron et al., 2009). The presence of untagged channels, however, could feasibly compensate for or obfuscate any functional deficits in our tests. Future studies will be necessary to confirm normal BK currents in various cell types. While FAP-BKα tagged channels do not label all channels, they effectively tetramerize with untagged channels, resulting in a plurality of tagged channels, which appear to have similar localization to untagged channels and can thus be used as detectable fluorescent reporters without overexpression.

### BKα May Have Tissue-Specific N-termini

BK channels exhibit vast variation in biophysical properties, in part due to extensive alternative splicing of BKα; this makes biologically relevant experiments by cDNA-based overexpression difficult. Multiple splice variants are expressed within a given tissue, with tissue-specific isoforms (Poulsen et al., 2009), which can form heterotetramers (Ma et al., 2007; Chen L. et al., 2010). Given our removal of upstream start sites, it was a surprise that untagged BKα persisted even in the FAP-BKα homozygotes. We did not identify a detectable cleavage of FAP from BKα; we reasoned that there could either be a rapid degradation of a cleaved FAP tag, or that translation start selection occurs, a phenomenon that has been observed in several potassium channels (Fernandez et al., 2003; Simkin et al., 2008). Interestingly, our data indicated that this effect is tissue specific, as this untagged variant was not observed in bladder smooth muscle. Additionally, the brain-specific putative untagged 125 kDa band in FAP-BKα Hom appeared narrowed compared to WT and FAP-BKα Het mice; however, the resolution was insufficient for reliable quantification of this phenomenon. Since BKα is not subject to extensive glycosylation (Hagen and Sanders, 2006) and considering the strategy for generation of the transgene, the missing upper component to this band could correspond to variants produced from the deleted MANG-translation start site.

Based on our Western blots, we concluded that in whole brain, about 25% of BKα included the FAP in Het, and 50% included the FAP in Hom mice, showing a genetic dose-dependence of expression. Interestingly, it appeared that FAP-BKα Hom had a weakened dendritic profile in PCs. Whether the FAP impairs dendritic trafficking or if the extended N-terminus that was removed in the FAP-BKα Hom mice is required for proper localization in PCs is an open question. This effect appeared specific to PCs; FAP signal in hippocampus showed prominent localization to distal processes in stratum oriens, stratum lacunosum-moleculare, and mossy fibers, but low expression in stratum pyramidale, as previously described (Misonou et al., 2006). Despite this change in BK localization in FAP-BKα homozygous mice, our measurements identified no motor defects. It intuits that the effects of altered subcellular BK localization would be more subtle than outright loss; this is evidenced by a lack of motor phenotype in β4 knockout mice (Brenner et al., 2005).

This mouse model, however, is not without limitations. Most notably, the unsuccessful characterization of the 5′-end of the insertion leaves open the possibility that regulatory elements crucial for BKα expression or splicing are altered; potentially producing abnormal BKα variants or misexpression in the brain. Immunostaining and Western blotting both showed decreased BKα signal in the brain, particularly in the homozygous mouse, which could potentially be due to unidentified changes in the vicinity of the targeted locus. An additional consideration is that a detailed transcript analysis was not successful, so while the inclusion of FAP in multiple splice isoforms is expected based on the intact *Kcnma1* locus 3′ of the insertion, it was not measured directly. If there is non-native regulation at play, the use of FAP-BKα Het mice can obviate these issues, and have FAP act as a passenger on otherwise normal heteromeric channels.

### β4 Regulation of Trafficking Is Not Limited to ER Retention

The β4 subunit has been described to restrict surface expression of BK channels in heterologous cells and CA3 pyramidal cells by the action of a novel ER retention motif (Shruti et al., 2012; Cox et al., 2014). This effect is dependent on cell type, with dentate gyrus granule cells and PCs natively exhibiting a population of surface BKαβ4 channels (Brenner et al., 2005; Benton et al., 2013). It seems likely that specific machinery or receptors are required for forward trafficking of β4 containing channels, as β4 is localized specifically to axons in several neuron types (Dopico et al., 1999; Misonou et al., 2006; Wynne et al., 2009) and exclusively to somatodendritic compartments in another (Martin et al., 2004). β4 is also crucial for localizing BK channels to apical membranes in the collecting duct of the kidney (Wen et al., 2013). It may be that ER retention is the dominant mode in heterologous cells where the requisite forward trafficking machinery for BKαβ4 channels is lacking (Shruti et al., 2012). In cells that show ER retention, such as CA3 pyramidal cells, it is feasible that specific inputs could release this pool.

Our work adds to the body of evidence for a cell-type-specific role of β4 in localizing surface BK channels. In PCs, β4 functions to facilitate channel localization to large somatic channel clusters, a phenomenon previously observed by electron microscopy (Kaufmann et al., 2009; Indriati et al., 2013). These previous studies determined a density of nine clusters per 100 μm^2^; envisioned as a 10 μm diameter sphere, this would be roughly 113 clusters per soma. Our results identifying 123 puncta per soma seem remarkably consistent with this. It is unknown how these channel clusters are formed, but the knockout of individual cluster components such as Ca_v_2.1, SK2, or BK channels, was shown to reduce cluster density (Indriati et al., 2013). A question to explore is whether β4 loss reduces the total number of clusters, or if it simply restricts BK channel incorporation. Unfortunately, our experimental workflow did not quantify β4 WT mice; it is possible that with WT levels of β4, more clusters would appear and enable us to examine a dosage relationship of β4 on BK channel clustering. Future experiments will aim to address this question. It is also possible that β4 knockout produces an enhancement of diffuse surface BK signal, but this was unclear in our system and will likely require dissociated cultures. While PCs are not alone in possessing clustered BK channels (Kaufmann et al., 2009, 2010), they do contain the largest clusters. The mechanisms of β4-directed localization to clusters and other PM domains in PCs and other neurons remain an enticing line of questions that may be answered using this model. Future studies will aim to address these questions and determine the roles that slow gated, β4-containing BK channels, or fast, β4-lacking BK channels may play in these domains to regulated neuronal function.

### Uses of the FAP-BKα Model

The far-red excitation and emission of the FAP-MG complex is useful for imaging in fixed tissue and does not require the long process of immunofluorescence for detection; furthermore, the far red spectrum leaves plenty of bandwidth for shorter wavelength single photon and two-photon probes. Non-specific fluorogen activation in nucleus posed an occasional problem in fixed tissue, an effect seen previously with labeling of dead cell nuclei (Yan et al., 2015). While development of MG-TCarb greatly reduced this issue, it did occasionally appear; we were unable to identify any particular condition with fixation or sectioning that contributed to this, but it was most present in WT brains where dye is not sequestered by its specific binding. In our validation of MG-TCarb labeling specificity compared to the HA tag, we found some regions where the intensities of HA labeling and MG-TCarb labeling do not linearly co-vary, likely due to differences in the detection method. Immunostaining could show variation in brightness based on several factors including accessibility of the epitope to primary antibody, the number of secondary antibodies bound to the primary for signal amplification, and binding specificity. On the other hand, MG-TCarb will always bind at most one FAP, resulting in a single fluorophore per tagged subunit, with increased access by virtue of being a small molecule.

Fluorogen-activating peptides exhibits its greatest strength in living cells, where charge-based cell impermeability can enable the visualization of exclusively surface proteins (Pratt et al., 2015; Yan et al., 2015). Our attempts to visualize FAP in acute living slices were hampered by non-specific contributions from dying cells, but this could be applied to organotypic slice cultures, where the cell exclusion is intact and the far-red spectrum enables tissue penetration. As an important factor for these future experiments, we confirmed that the cell-excluded MG-BTau dye does not in itself change any of the electrical properties of neocortical neurons. Because aberrant BK trafficking may underlie kindling and seizure sensitization (Shruti et al., 2008), future experiments are aimed at examining dynamic regulation of BK channel trafficking in dissociated cultures taken from this model. Activity-dependent regulation of channel localization has been observed in other potassium channels (Kim J. et al., 2007; Connors et al., 2008). Dissociated neurons and organotypic explants derived from the FAP-BKα Het mouse line would contain tagged channels under near native expression levels, results gained would not be subject to overexpression artifacts, and preservation of native splicing control would allow focus on the most relevant isoforms, even if not every channel is tagged. The FAP system is sufficiently bright and photostable for single-molecule tracking (Saurabh et al., 2016). Live cell experiments examining channel redistribution are possible in this biologically relevant context with proper BKα stoichiometry with β and γ subunits.

## Ethics Statement

All experiments were approved by the Institutional Animal Care and Use Committee of Carnegie Mellon University.

## Author Contributions

CP, MB, and AB designed research. CP and DAK performed research and analyzed data. GH and CP designed CRISPR. SD and MH performed electrophysiology to characterize FAP-BKα properties. JH and DK designed and synthesized MG-TCarb.

## Conflict of Interest Statement

MB is a founder in Sharp Edge Labs, Inc., a company exploiting the FAP-Fluorogen tagging for drug discovery. The other authors declare that the research was conducted in the absence of any commercial or financial relationships that could be construed as a potential conflict of interest. The reviewer CC declared a past co-authorship with one of the authors AB to the handling Editor.

## Footnotes

1 http://www.antibodiesinc.com
