# Supplementary material for: Tagging of Endogenous BK Channels with a Fluorogen-Activating Peptide Reveals β4-Mediated Control of Channel Clustering in Cerebellum
Source: Front Cell Neurosci. 2017 Oct 31;11:337. doi: 10.3389/fncel.2017.00337 (PMC5671578; doi:10.3389/fncel.2017.00337)
Supplement: Supplementary file 1 [file Data_Sheet_1.PDF]

Supplementary Material

Tagging of Endogenous BK Channels with a Fluorogen-Activating Peptide Reveals β4-Mediated Control of Channel Clustering in Cerebellum

Christopher P. Pratt[1,3,4], Dika Kuljis[1], Gregg E. Homanics[5], Jianjun He[2,3], Dmytro Kolodieznyi[2,3], Srikanth Dudem[6], Mark A. Hollywood[6], Alison L. Barth[1,4], Marcel P. Bruchez[1,2,3]

[1] Department of Biological Sciences, [2] Department of Chemistry, [3] Molecular Biosensor and Imaging Center, [4] Center for the Neural Basis of Cognition, Carnegie Mellon University, Pittsburgh PA

[5] Departments of Anesthesiology, Neurobiology, and Pharmacology & Chemical Biology, University of Pittsburgh, Pittsburgh PA

[6] Smooth Muscle Research Centre, Dundalk Institute of Technology, Dundalk Ireland

* **Correspondence:** Marcel P. Bruchez: bruchez@cmu.edu

1 Supplementary Methods

1.1 Generation of dL5**-BK α Mice

CRISPR/Cas9 and Repair Template Production: Two sgRNAs targeting the MANG and MDAL transcription start sites in exon 1 (sgRNA#1 and sgRNA#2, respectively; **see Fig. 2A** in main text) of *Kcnma1* were identified using the CRISPR Design Tool (Hsu et al., 2013). Unique PCR primers (See Table S1) that contained the sgRNA target site and overlapped with a common reverse primer were used to generate T7 promoter containing sgRNA templates for each site as described (Bassett et al., 2013). These templates were transcribed in vitro using a MEGAshortscript Kit (Ambion). The Cas9 coding sequence was amplified from pX330 (Cong et al., 2013) using a T7 promoter containing forward primer and reverse primer (Table 1) and subcloned into pCR2.1-TOPO. This plasmid was linearized with EcoRI, in vitro transcribed and polyA tailed using the mMessage mMachine T7 Ultra Kit (Ambion). Following synthesis, the sgRNA and Cas9 mRNA were purified using the MEGAclear Kit (Ambion), ethanol precipitated, and resuspended in DEPC treated water. A 5300 bp *Kcnma1* targeting plasmid containing the 855 bp insertion with the fluorogenic tag and 800 bp of homology to *Kcnma1* exon 1 was synthesized (Genewiz, South Plainfield, NJ). Plasmid DNA was purified with Plasmid Midi Kit (Qiagen), ethanol precipitated, and resuspended in TE.

Mouse Production: sgRNA (12.5-60 ng/µl), Cas9 mRNA (30-110 ng/µl), and repair plasmid (100-360 ng/µl) were combined in embryo injection buffer (10mM Tris, pH 7.4, 0.1mM EDTA), aliquotted, and stored at -80 C until use. C57BL/6J one-cell embryos were collected from superovulated females and cultured in KSOM at 37C in 5% CO_2 / 95% air. Embryos were briefly transferred to M2 medium and the nucleic acid mixture was injected into the pronucleus or cytoplasm as described (Yang et al., 2014). Embryos that survived injection were transferred to the oviduct of day 0.5 postcoitum pseudopregnant CD-1 recipient females. Pups resulting from injected embryos were screened for targeting of the *Kcmna1* gene by PCR with a variety of PCR primer sets. For example, a 263 bp amplicon that is unique to a targeted *Kcmna1* locus was amplified with primers (BK F2, BK R8; Table 1) that bind to the dL5** tag and *Kcnma1* intron 1. All samples were also amplified with a control primer set (RhoI F1, R1; Table 1) to verify the integrity of the DNA preparations. To confirm the fidelity of the targeting amplicon, PCR products were sequenced directly or subcloned into pCR2.1-TOPO (Invitrogen) and sequenced.

Mouse genomic tail DNA was also analyzed by Southern blot analysis to confirm targeting at the BK ✔ locus and to investigate the structure of the targeted locus. Genomic DNA was digested with EcoRV or BglII, run on 1% agarose gels, transferred to Hybond (GE Healthcare Life Sciences), and subsequently hybridized to 5' and 3' probes that were external to the targeting construct. The 5' probe was a 542 bp PCR product (F primer: 5'-TCTGTGTGCAACGATAGGGG-3'; R primer: 5'-TAGGCCGGTTTTCCCAAAGG-3'). Two 3' probes were used: a 586 bp PCR product (F primer: 5'-TCCAAGGGACCTCTGCTTCA-3'; R primer: 5'-CCACAAAACACAGCTGAGCC-3') and a 401 bp PCR product (F primer: 5'-CCCCAAATGTTCACACCTGC-3'; R primer: 5'-GGAAGGCCATCGGTCTTTCT-3').

Initial Mouse Genotype Analysis: For initial screening genotype analysis, a crude DNA extract was prepared from mouse tail tips or ear punches using 150 µl QuickExtract (Epicentre, Madison, WI). The targeted *Kcnma1* and control *RhoI* amplicons were amplified with PCR as described above. PCR products were analyzed on 2% agarose in TAE buffer.

1.2 Synthesis of MG-TCarb

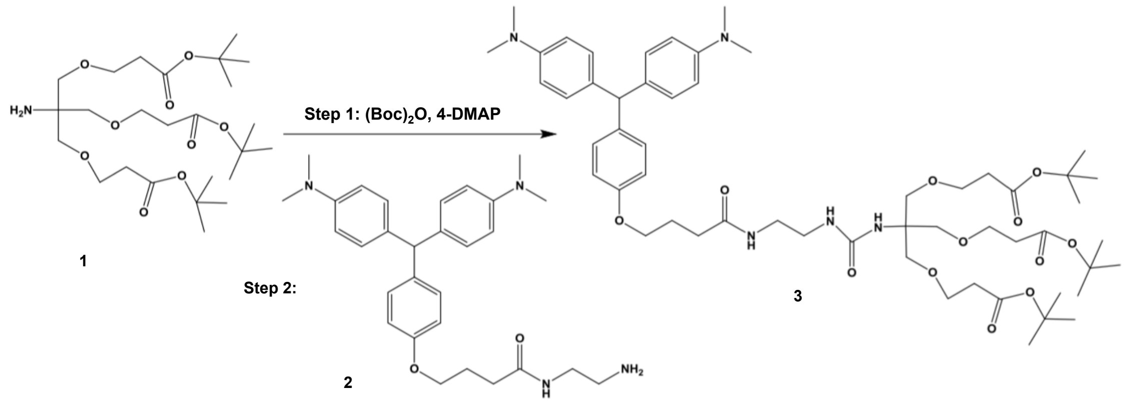

0.917 g (4.2 mmol) di-tert-butyldicarbonate ($(Boc)_2O$) and 0.368 g (3 mmol) 4-DMAP were dissolved in 10 mL dry CH_3CN. 1.48 g (3 mmol) tripod (Structure 1, synthesized and provided by Dr. Brigitte Schmidt (Szent-Gyorgyi et al., 2010)) was dissolved in 10 mL dry CH_3CN and added. The mixture was stirred for 10 minutes at room temperature. 1.42 g (3 mmol) MG[H]-EDA (Structure 2, routinely synthesized following the literature (Szent-Gyorgyi et al., 2007)) was dissolved in 25 mL CH_3CN and added to the reaction mixture. After stirring at room temperature for 5 hours, thin-layer chromatography (TLC) was used to detect reaction completion. The reaction mixture was dried under reduced pressure and purified with silica gel after ethyl acetate elution (KnDIN ZOTERO_ITEM CSL. The product (Structure 3) had a chemical composition of $C_{55}H_{83}N_5O_{12}$ with M.W. of 1005.60 g/mol (yield: 2.25 g, 74%). ^1H NMR (500 MHz, Methanol-d4) δ 7.33 (d, J = 9.0 Hz, 4H), 7.27 (d, J = 9.2 Hz, 2H), 7.08 (d, J = 8.8 Hz, 2H), 6.95 (d, J = 9.1 Hz, 4H), 5.52 (s, 1H), 4.11 (t, J = 6.3 Hz, 2H), 3.53 (m, 12H), 3.22 (s, 12H), 3.14 (t, J = 6.1 Hz, 2H), 3.08 (t, J = 6.5 Hz, 2H), 2.34 (m, 8H), 2.06 (m, 2H), 1.35 (s, 27H). ^{13}C NMR (125 MHz, Methanol-d4) δ 172.8, 171.3, 158.8, 157.0, 148.9, 137.6, 133,3, 130.2, 129.9, 114.1, 112.6, 80.7, 70.1, 67.2, 58.9, 54.2, 41.8, 40.8, 39.4, 36.4, 36.2, 31.0, 28.1, 25.4. ESI-MS (+m/z): 1004.5.

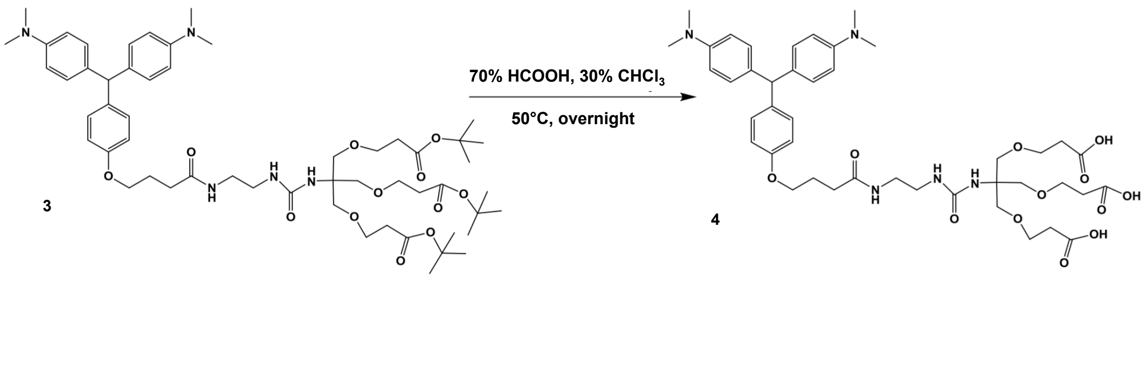

2.01g (2 mmol) MG[H]-EDA-Tripod (Structure 3) was dissolved in 10 mL $HCOOH/CHCl_3$ in a 4:1 ratio and heated to 50°C overnight. The solvent was removed under reduced pressure; raw product was purified using a silica gel column with an 80%/20% mixture of $CHCl_3$/MeOH as eluent (Han et al., 2001). The product (Structure 4) had a chemical composition of $C_{43}H_{59}N_5O_{12}$ with M.W. of 837.97 g/mol (yield: 1.51g, 91%). ^1H NMR (500 MHz, Methanol-d4) δ 7.31 (d, J = 8.7 Hz, 4H), 7.23 (d, J = 9.2 Hz, 4H), 7.02 (d, J = 9.0 Hz, 2H), 6.86 (d, J = 9.3 Hz, 2H), 5.58 (s, 1H), 4.01(t, J = 6.0 Hz, 2H), 3.67 (m, 12H), 3.33 (t, J = 6.3 Hz, 2H), 3.22 (t, J = 6.1 Hz, 2H), 3.16 (s, 12H), 2.52 (m, 6H), 2.39 (t, J = 6.6 Hz, 2H), 2.08 (m, 2H). ^{13}C NMR (125 MHz, Methanol-d4) δ 174.4, 174.1, 159, 157.8, 144, 142, 135.4, 130.5, 130, 117.8, 114.2, 69.8, 66.8, 58.9, 57, 54.7, 43.9, 39.9, 38.7, 34.4, 32.2, 25.3, 17.1. ESI-MS (+m/z): 838.3.

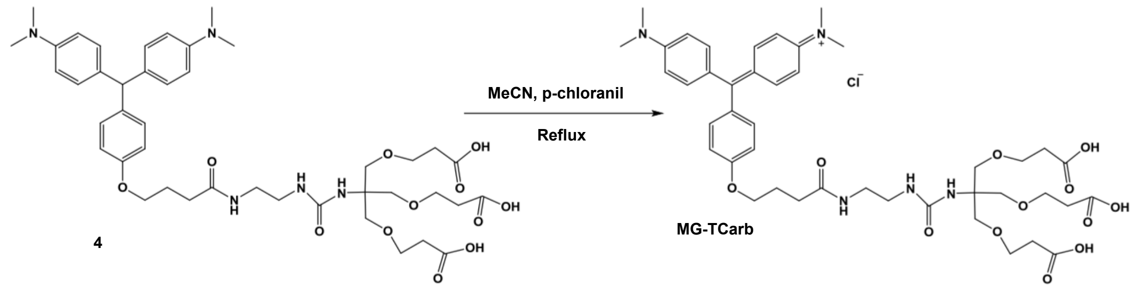

0.838 g (1 mmol) MG[H]-EDA-$COOH_3$ (Structure 4) was dissolved in MeCN and heated to reflux. 0.271 g (1.1 mmol) p-chloranil was dissolved in hot MeCN and added to the reaction; the reaction mixture was refluxed for an additional 2-3 hours. The reaction mixture was dried under reduced pressure and purified using a silica gel column with 80% $CHCl_3$/20% MeOH eluent. The product (MG-TCarb) had a chemical composition of $C_{43}H_{58}ClN_5O_{12}$ with M.W. of 872.41 g/mol (yield: 0.763 g, 88%). ^1H NMR (500 MHz, Methanol-d4) δ 7.60 (d, J = 8.9 Hz, 4H), 7.54 (d, J = 8.3 Hz, 2H), 7.38 (d, J = 8.7 Hz, 2H), 7.21 (d, J = 9.0 Hz, 4H), 4.43 (t, J = 6.2 Hz, 2H), 3.93 (t, J = 7.2 Hz, 6H), 3.86 (s, 6H), 3.53 (s, 12H), 3.48 (t, J = 6.1 Hz, 2H), 3.41 (t, J = 5.9 Hz, 2H), 2.80 (t, J = 6.0 Hz, 6H), 2.68 (t, J = 6.3 Hz, 2H), 2.38 (p, J = 6.8 Hz, 2H). ^{13}C NMR (125 MHz, Methanol-d4) δ 177.3, 175.8, 175.5, 164, 159.5, 157.1, 141, 138, 132.3, 126.9, 119.4, 115.2, 113.7, 69.9, 68.3, 67.2, 40.7, 40.2, 39.1, 35, 32.8, 25. ESI-MS (+m/z): 836.3.

1.3 Improved Synthesis of MG-TCarb

The synthetic procedure described above was used for all experiments; however, it resulted in relatively low yield and purity of the final product compared to other dyes synthesized in house. We detail here optimizations in synthesis that have resulted in higher purity and yield:

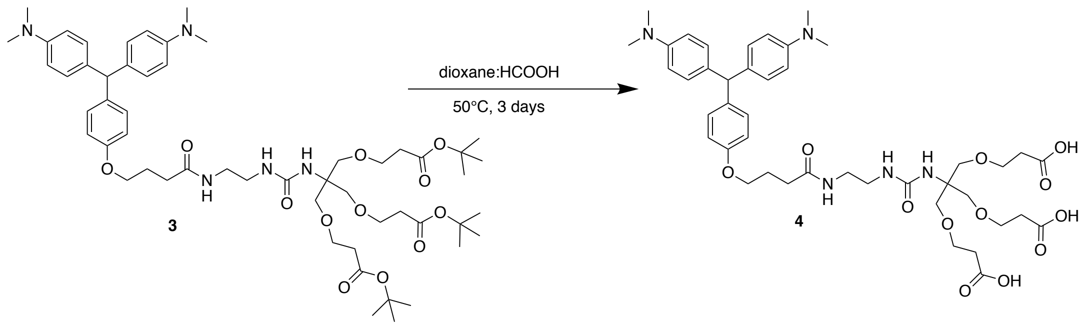

For the deprotection step, solvent was changed to 10 mL HCOOH/dioxane3 in a 7:3 ratio and stirred at 50°C for 3 days. The solvent was removed under reduced pressure; raw product was purified using a silica gel column with gradient elution using hexane:ethyl acetate. The product (Structure 4) had a chemical composition of $C_{43}H_{59}N_5O_{12}$ with M.W. of 837.97 g/mol. ^{1}H-NMR spectrum matches that previously reported.

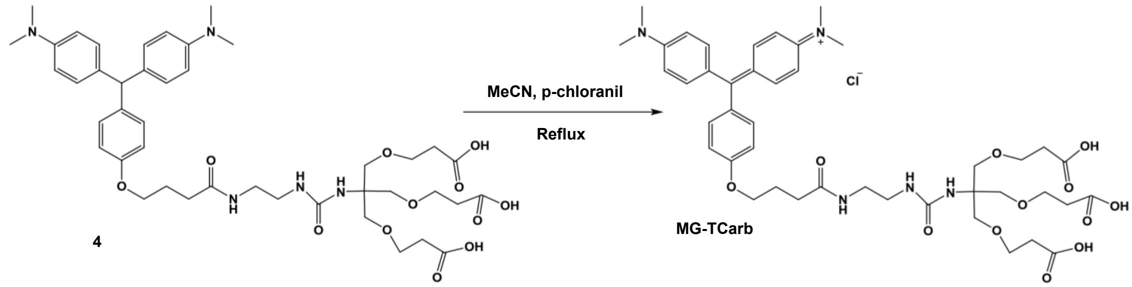

For the oxidation step, the main change is how the purification and the workup after the reaction is done: reaction mixture was dried under reduced pressure and purified using reverse-phase chromatography (C4 cartridge from Biotage; water (1% ammonia):methanol gradient). The product (MG-TCarb) had a chemical composition of $C_{43}H_{58}ClN_5O_{12}$ with M.W. of 872.41 g/mol. ESI-MS (+m/z): 836.3. ^{1}H-NMR spectrum matches that previously reported.

2 Supplementary Figures and Tables

Primer	Sequence (5'-3')
**MANG Forward**	GAAATTAATACGACTCACTATAGGCTGCCGCCCATTGCTAGCTAGTTTTAGAGCTAGAAATAGC
**MDAL Forward**	GAAATTAATACGACTCACTATAGGCTCGGTCCACGAGCCCAAGAGTTTTAGAGCTAGAAATAGC
**Common Reverse**	AAAAGCACCGACTCGGTGCCACTTTTTCAAGTTGATAACGGACTAGCCTTATTTTAACTTGCTATTTCTAGCTCTAAAAC
**T7-Cas9 Forward**	TATTACGACTCACTATAGGGAGAATGGACTATAAGGACCACGAC
**Cas9 Reverse**	GCGAGCTCTAGGAATTCTTAC
**BK F2**	ACATGGCTGTTGATGGGTGT
**BK R8**	TACCACCTGGGCTAACGGTA
**RhoI F1**	AGATGAGGGGCAGAGAACCT
**RhoI R1**	CTTCTCCCGAAGCTTCCGTT
**Genotyping Fw**	GCAACATGGCTGTTGATGGGTGTTC
**Genotyping Rev**	GTCACCGGTATGATGAGCGCATCC

Table S1: PCR Primers used for verification and genotyping. sgRNA target sites are underlined. Bold sequences show T7 promoter sequence

2.1 Supplementary Figures

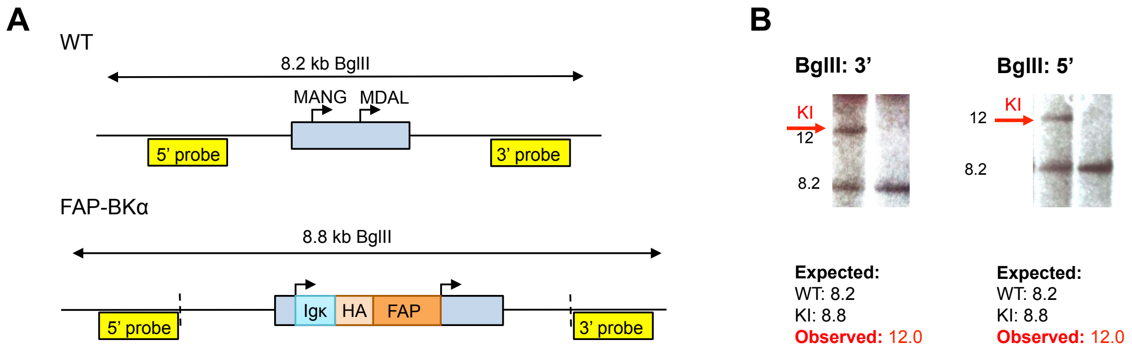

Figure S1: BglII Southern Blots (A) Schematic of Southern Blotting with expected digestion fragment shown **(B)** BglII Southern Blots with 3' and 5' external probes show a larger than expected 12 kb band.

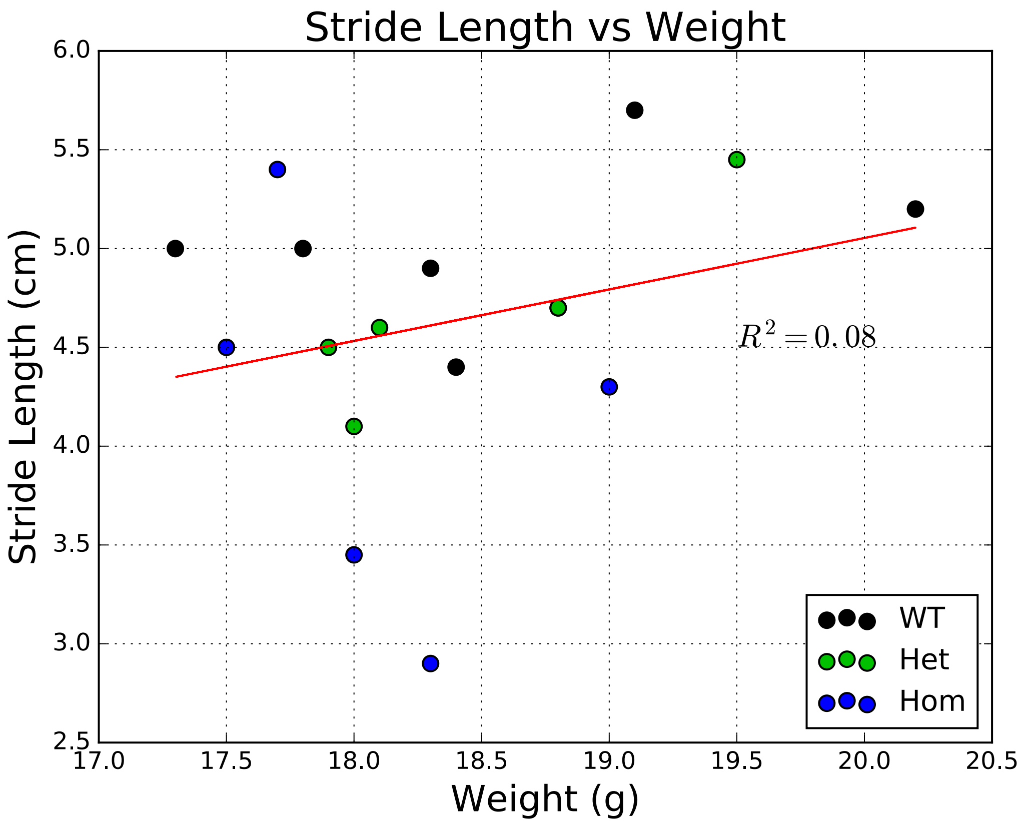

Figure S2: Stride length weakly scales with weight: Weights and stride length were plotted for 17 animals (WT 4x F, 2x M; Het 5x F, 1x M; Hom 4x F, 1x M). Linear regression (red line) and R^2 value represent the aggregate of all data across genotypes. The predictive value of weight on stride length is negligible, due to the large individual variation in stride length. The two Hom mice with the shortest stride appear as outliers; these were female and did not show any deficit in coordination in the rotarod test.

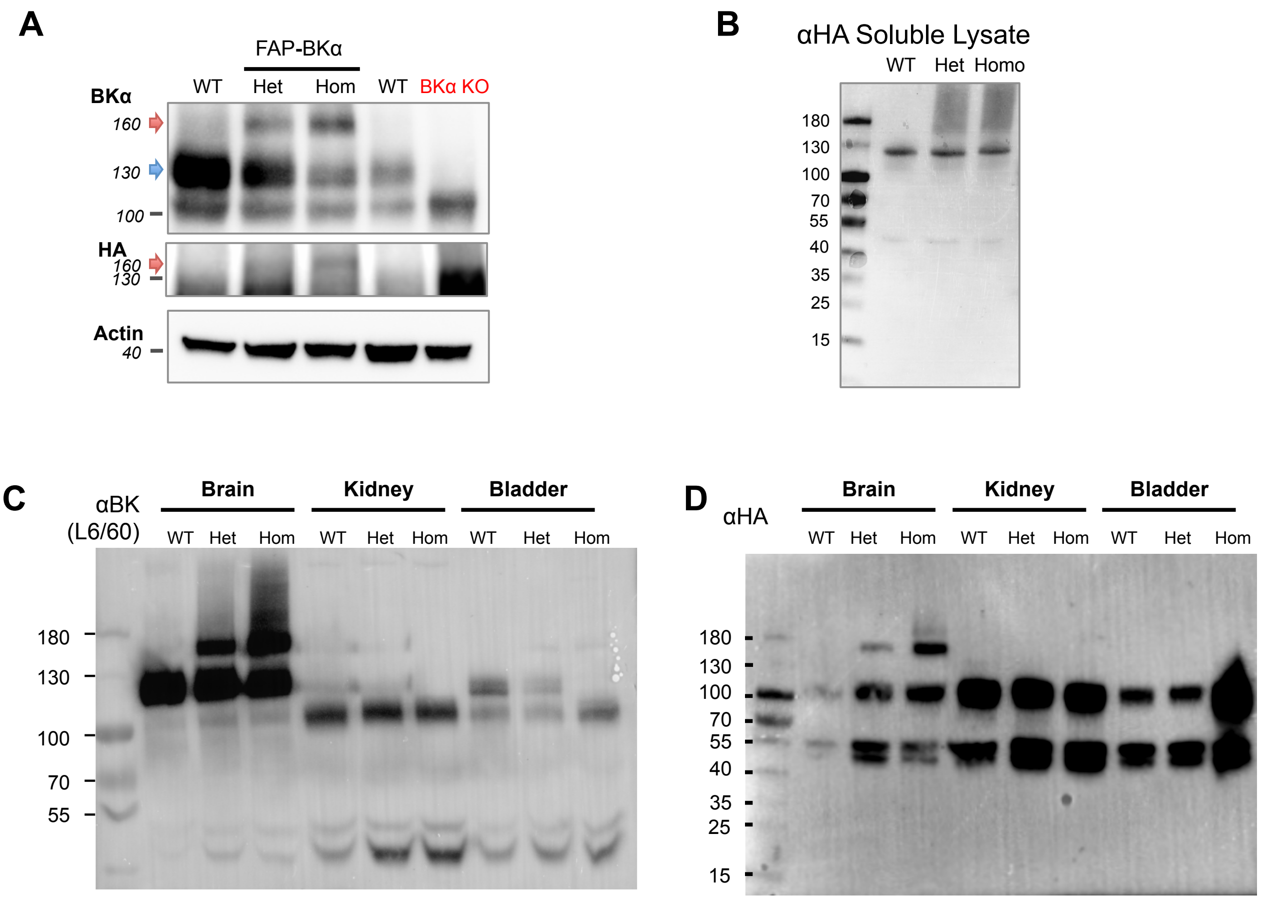

Figure S3: BKα antibody is specific, no HA-reactive cleavage products are detectable in membrane and whole lysates (A) FAP-BKα Het and Hom brains retain untagged BKα. BKα KO lysates demonstrate specificity of the BKα antibody. The 130 kDa band disappears in BKα KO, showing that this band in FAP-BKα Hom is specific. **(B)** Gradient SDS-PAGE of whole brain lysate probed for HA shows no specific low molecular weight products, suggesting that any cleavage products are either short-lived or absent. **(C-D)** Full blots of membrane fractionated brain, kidney, and bladder lysates including M.W. ladder. Probing for BKα (C) or HA (D) shows nonspecific bands at approximately 100 kDa. HA blotting shows a lack of detectable membrane-bound cleavage products.

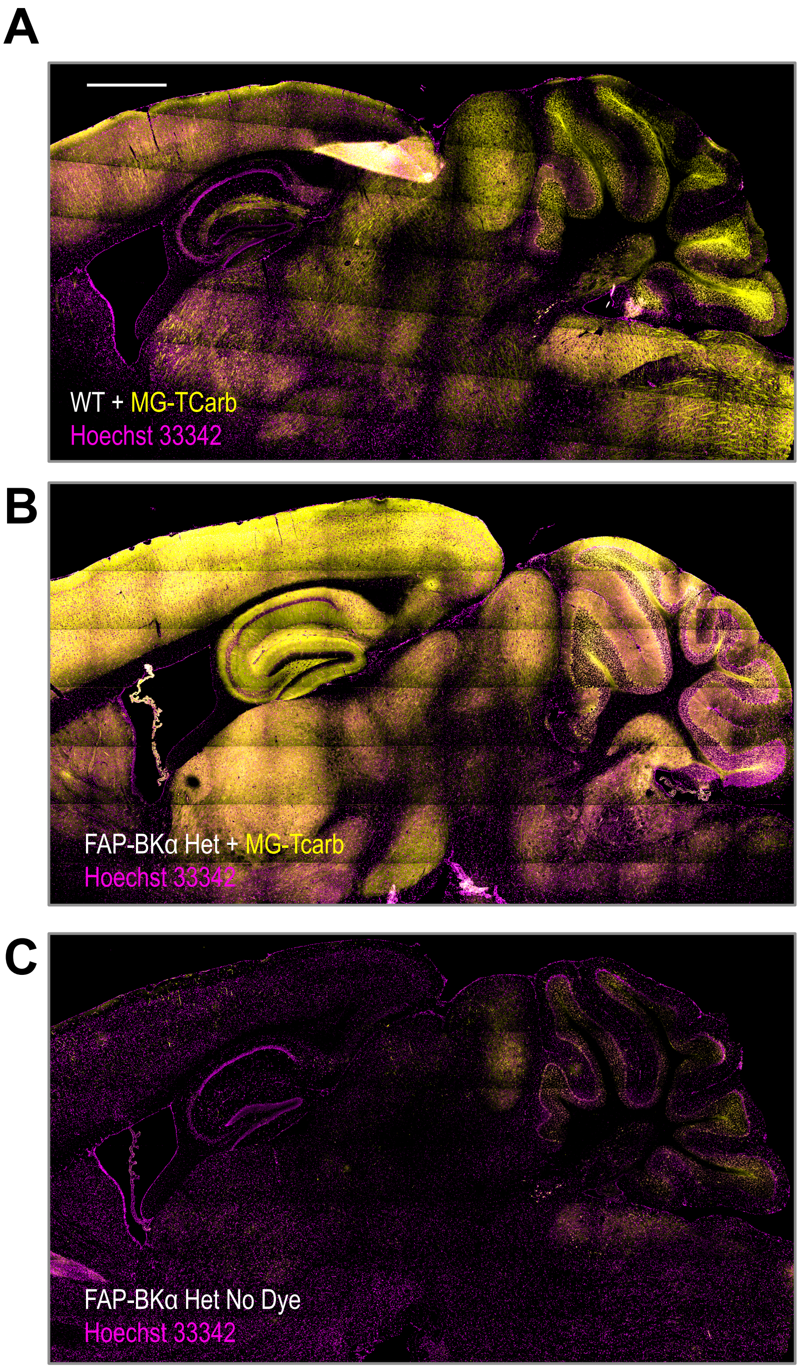

Figure S4: Sagittal sections of FAP-BK α Het and WT brain shows specificity of FAP+MG-TCarb labeling. (A) WT brain subjected to MG-TCarb labeling shows mild dye-related background signal in cerebellar granule cell layers, fiber tracts, and hindbrain. Hoechst 33342 staining is used for better landmark visibility. **(B)** Treatment of FAP-BK α Het brain with MG-TCarb shows bright specific fluorescence in hippocampal formation, cortex, cerebellar PCL and ML, among others, mirroring BK α distribution in Figure 5. **(C)** Omission of MG-TCarb labeling protocol results in a lack of fluorescence in FAP-BK α Het brain sections. Scale bar 1 mm.

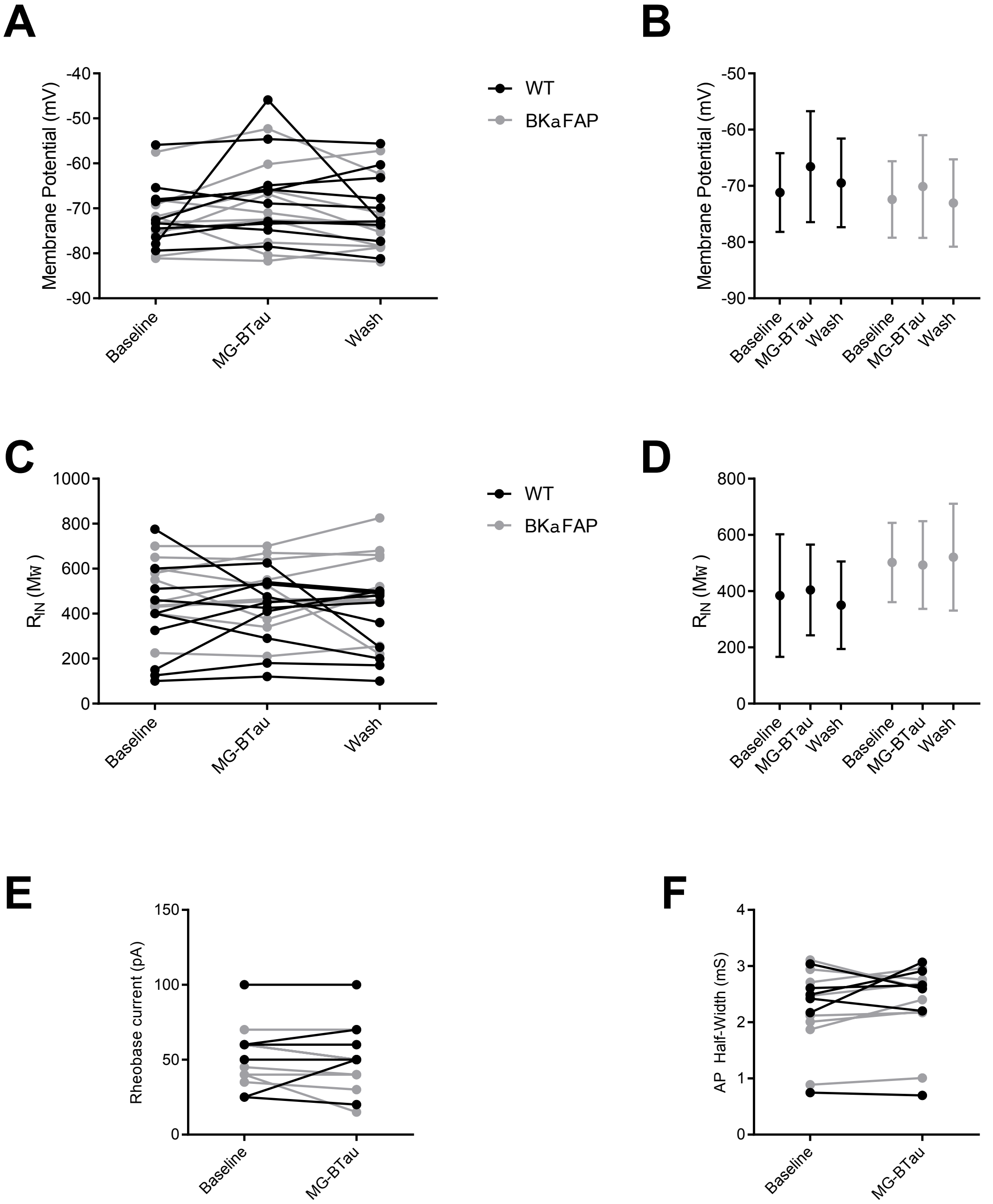

Figure S5: MG-BTau does not adversely affect electrical properties of neocortical neurons (A) Resting membrane potential before, during, and after MG-BTau perfusion. **(B)** Summary data from A. **(C)** Input resistance (R_{IN}) before, during, and after MG-BTau perfusion. **(D)** Summary data from C. **(E)** Rheobase current is elicited before MG-BTau addition and after MG-BTau addition. **(F)** AP half-width without and with MG-BTau in perfusate. *n* = 10 mice per genotype. Zygosity of FAP-BK α was undetermined at the time of these experiments.

3 References

Bassett, A. R., Tibbit, C., Ponting, C. P., and Liu, J.-L. (2013). Highly efficient targeted mutagenesis of Drosophila with the CRISPR/Cas9 system. *Cell Rep.* 4, 220–228. doi:10.1016/j.celrep.2013.06.020.

Cong, L., Ran, F. A., Cox, D., Lin, S., Barretto, R., Habib, N., et al. (2013). Multiplex genome engineering using CRISPR/Cas systems. *Science* 339, 819–823. doi:10.1126/science.1231143.

Han, G., Tamaki, M., and Hruby, V. j. (2001). Fast, efficient and selective deprotection of the tert-butoxycarbonyl (Boc) group using HCl/dioxane (4 m). *J. Pept. Res.* 58, 338–341. doi:10.1034/j.1399-3011.2001.00935.x.

Hsu, P. D., Scott, D. A., Weinstein, J. A., Ran, F. A., Konermann, S., Agarwala, V., et al. (2013). DNA targeting specificity of RNA-guided Cas9 nucleases. *Nat. Biotechnol.* 31, 827–832. doi:10.1038/nbt.2647.

Knölker, H.-J., Braxmeier, T., and Schlechtingen, G. (1995). A Novel Method for the Synthesis of Isocyanates Under Mild Conditions. *Angew. Chem. Int. Ed. Engl.* 34, 2497–2500. doi:10.1002/anie.199524971.

Szent-Gyorgyi, C., Schmidt, B. A., Creeger, Y., Fisher, G. W., Zakel, K. L., Adler, S., et al. (2007). Fluorogen-activating single-chain antibodies for imaging cell surface proteins. *Nat. Biotechnol.* 26, 235–240.

Szent-Gyorgyi, C., Schmidt, B. F., Fitzpatrick, J. A. J., and Bruchez, M. P. (2010). Fluorogenic dendrons with multiple donor chromophores as bright genetically targeted and activated probes. *J. Am. Chem. Soc.* 132, 11103–11109. doi:10.1021/ja9099328.

Yang, H., Wang, H., and Jaenisch, R. (2014). Generating genetically modified mice using CRISPR/Cas-mediated genome engineering. *Nat. Protoc.* 9, 1956–1968. doi:10.1038/nprot.2014.134.
